# Signatures of local nitrogen adaptation in the *Brachypodium distachyon* root microbiome

**DOI:** 10.1111/nph.70684

**Published:** 2025-10-29

**Authors:** Kevin D. Ricks, Sierra S. Raglin, Angela D. Kent

**Affiliations:** ^1^ Department of Ecology and Evolutionary Biology University of Toronto Toronto ON M6G 3G2 Canada; ^2^ Program in Ecology, Evolution, and Conservation Biology University of Illinois Urbana‐Champaign Urbana IL 61801 USA; ^3^ Department of Natural Resources and Environmental Sciences University of Illinois Urbana‐Champaign Urbana IL 61801 USA; ^4^ Carl R. Woese Institute for Genomic Biology University of Illinois Urbana‐Champaign Urbana IL 61801 USA; ^5^ DOE Center for Advanced Bioenergy and Bioproducts Innovation University of Illinois at Urbana‐Champaign Urbana IL 61801 USA

**Keywords:** *Brachypodium distachyon*, evolutionary ecology, local adaptation, microbiomes, nitrogen cycling, plant–microbe interaction, rhizosphere, symbiosis

## Abstract

- Plants associate with diverse microbiomes that impact their fitness, yet the contribution of the microbiome to plant adaptation is uncertain. As plant recruitment of its microbiome can be both highly variable and genetically determined, we hypothesized this recruitment process may be the result of adaptive evolution, and contributing to plant local adaptation.
- We investigated the evolution and adaptive benefit of plant–microbiome recruitment by characterizing the rhizosphere communities across a genotypic panel of *Brachypodium distachyon* in a common garden experiment. By linking microbial communities to their host genotype's historic environment, we identified signatures of selection on plant–microbiome recruitment.
- Plant–microbiome composition was significantly correlated with the host genotype's historic environment, with enrichment of microbial traits aligned to local resource conditions. For example, genotypes from low‐nitrogen environments recruited communities enriched in nitrogen acquisition traits. In a complementary experiment evaluating plant nitrogen response, these same genotypes were well‐adapted to low‐nitrogen environments, contingent on the presence of key nitrogen‐cycling microbes.
- These results suggest that local adaptation in plants may partially be mediated by recruitment of beneficial microbiomes. This perspective suggests that plant adaptation may be an emergent property of host–microbe interactions, where evolutionary responses favor traits that promote recruitment of locally beneficial microbiomes.

## Introduction

While plants interact with complex microbial communities that influence their fitness and phenotype (Friesen *et al*., 2011; Trivedi *et al*., 2020), the contribution of these microbial assemblages to plant adaptation remains unclear. Microbiomes colonize every plant surface and can impact host fitness through specific functions, such as nutrient provisioning, immune system modulation, and pathogenesis (Friesen *et al*., 2011; Turner *et al*., 2013; Vandenkoornhuyse *et al*., 2015). As global environmental change accelerates, understanding how microbiomes mediate adaptive or maladaptive plant phenotypes will be increasingly important (Trivedi *et al*., 2022). Consequently, there has been significant interest in understanding the factors that shape microbiome assembly, as shifts in microbiome composition alter the availability of microbial traits and functions that interact with and influence plant fitness (Qu *et al*., 2020; Trivedi *et al*., 2020, 2022). Identifying the evolutionary and ecological drivers of these interactions and the distribution of microbial functions will enable predictions of plant responses to environmental change and strategies to harness microbiomes for improved plant resilience.

While microbiome assembly can be influenced by various drivers, including the abiotic environment, priority effects, and dispersal (Koide *et al*., 2017; Fitzpatrick *et al*., 2018; Leopold & Busby, 2020), the plant genetic background can also be a significant driver in these communities (Zhang *et al*., 2023). For example, plant diversity panels have highlighted large amounts of intraspecific variation in the microbiome (Compant *et al*., 2019; Leopold & Busby, 2020), while genome‐wide association studies and knockout experiments have identified specific genetic loci and plant traits that can alter microbiome structure (Wallace *et al*., 2018; Deng *et al*., 2021; Van Wallendael *et al*., 2022; Boyle *et al*., 2024). These traits influence microbiome recruitment by filtering the microbes that colonize and persist on the plant (Ricks & Koide, 2019). Boyle *et al*. (2024), for example, recently showed that leaf morphology can significantly alter microbiome composition, potentially through subtle changes to the abiotic environment surrounding the leaf, including temperature and humidity. Such changes effectively act as ecological filters, selecting for microbes that can tolerate these specific microenvironmental changes. A variety of other plant traits, including root physiology and exudation, internal chemistry, metabolite production, hormone signaling, immune function, and morphology, further contribute to the plant's recruitment of its microbiome (González‐Teuber *et al*., 2020; Eichmann *et al*., 2021; Seitz *et al*., 2022; Galindo‐Castañeda *et al*., 2024). The genetic basis of these drivers of plant–microbiome interactions makes the microbiome, to a certain degree, heritable (Peiffer *et al*., 2013; Deng *et al*., 2021; Boyle *et al*., 2024), and suggests that microbiome recruitment can evolve. As shifting environments pose novel pressures, we focus here on how microbiome recruitment may evolve: either as a direct adaptation to increase plant resilience or as a byproduct of other adaptations.

**Fig. 1:**
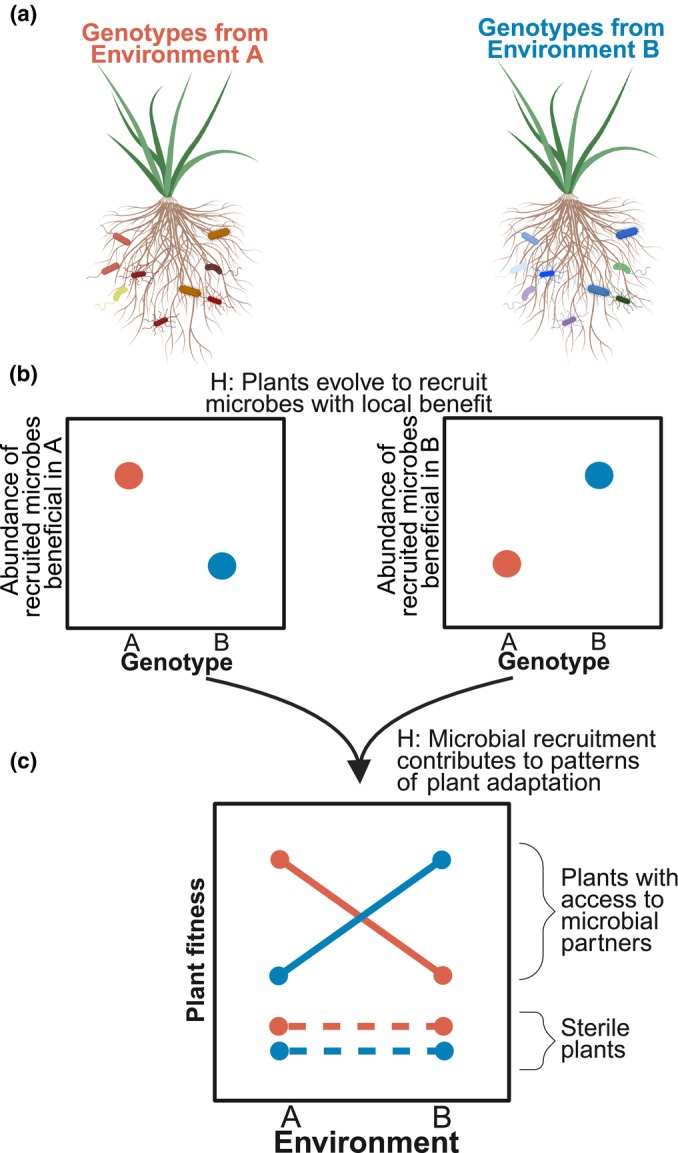
Expectations for adaptive recruitment in the microbiome. If plants evolve to recruit microbiomes with specific benefits, we should expect (a) plants from different environments to have unique microbiome compositions. Moreover, (b) compositional differences between these environments should be associated with microbial taxa that have specific fitness benefits in their home environment. A consequence of these recruitment patterns is that (c) plant local adaptation may be dependent on the presence of microbial partners to interact with. This figure was created in BioRender Raglin, S. (2025, BioRender.com/m47ab15).

Plant genetic drivers of microbiome recruitment may represent adaptive variation, with plants evolving traits to recruit optimal microbiomes for their native habitat (Fig. 1a,b). Because microbial associations can impose trade‐offs on plant performance, the optimal microbiome for a plant depends on its current environmental context (Rodriguez *et al*., 2008; Lau & Lennon, 2012). Consequently, there may be selection for host traits that promote the recruitment and colonization of specific microbes or microbial functions that improve plant fitness in a given environment. In this way, the plant genetic drivers influencing microbiome composition may represent pathways for plant adaptive evolution. The enormous diversity in plant microbiomes, both within and between host species (Turner *et al*., 2013; Vandenkoornhuyse *et al*., 2015), may in turn represent the outcome of these evolutionary processes and plant adaptation. Consistent with this, multiple lines of empirical work suggest plant adaptation can depend on the presence of microbial partners, although explicit microbial mechanisms underlying this adaptation have not been shown (Fig. 1c; Petipas *et al*., 2020; Ricks *et al*., 2023; Brady & Farrer, 2024).

Alternatively, microbiome recruitment may not necessarily represent adaptive variation as the plant traits impacting recruitment can be shaped by selective pressures beyond microbial interactions. Genetic variation in plant traits that indirectly alters microbiome recruitment may lead to colonization by microbiomes with neutral or maladaptive fitness effects. Consequently, the microbial community may merely be an incidental byproduct of plant adaptation, rather than the direct target of selection. For example, while plant hormonal expression can influence microbial recruitment (Eichmann *et al*., 2021), its evolution for reasons unrelated to microbial interactions can cascade to incidentally impact the microbiome. Such processes could lead to the recruitment of the ‘wrong’ microbes that are neutral or maladaptive for the environment, potentially slowing adaptive responses. Of course, adaptive and neutral variation in microbiome phenotypes are not necessarily mutually exclusive and could depend on the microbial functional group. Some plant traits may evolve to regulate microbial processes important for plant fitness, while variation in other microbial functional groups may be a byproduct of other plant adaptive phenotypes.

We investigated the potential for adaptive recruitment of the plant microbiome by conducting two glasshouse experiments using the model grass, *Brachypodium distachyon* (L.). First, we took a comparative analysis approach, characterizing rhizosphere microbiomes across a genotypic panel of *B. distachyon* grown in a common garden. As a portion of plant genotypic microbiome variation may be driven by abiotic selection, we could first characterize the end result of this selection by linking microbiomes to their host's historical environments (Endler, 1986). We hypothesized this genotypic variation was partially driven by plants evolving to adaptively recruit locally beneficial microbes, addressing this hypothesis by focusing on the communities of microbial nutrient cyclers. Microbial nutrient provisioning processes catalyze and transform the bioavailability of growth‐limiting nutrients (Miransari, 2013) and underlie many host–microbe symbioses (Martin *et al*., 2017). These groups are ideal systems for testing this hypothesis as nutrient stress strongly inhibits plant physiology, with tractable taxonomic groups modifying nutrient availability in the rhizosphere, including mycorrhizal fungi, nitrifying‐bacteria and archaea, nitrogen‐fixing bacteria, and phosphorus‐provisioning microorganisms (Miransari, 2013). We assessed adaptive recruitment in these groups by linking their recruitment with the plant's historical nutrient availability (Fig. 1b), as prior work in these nutrient cycling groups provides *a priori* hypotheses for locally beneficial assemblages (Jacoby *et al*., 2017). As recruitment of these groups may shape patterns of plant adaptation (Fig. 1c), we further tested these hypotheses by conducting a complementary experiment manipulating nitrogen availability and microbial associations, linking microbes to the plant nitrogen response.

## Materials and Methods

### Linking plant–microbiome structure with their historical environments

#### Genotype selection

**Fig. 2:**
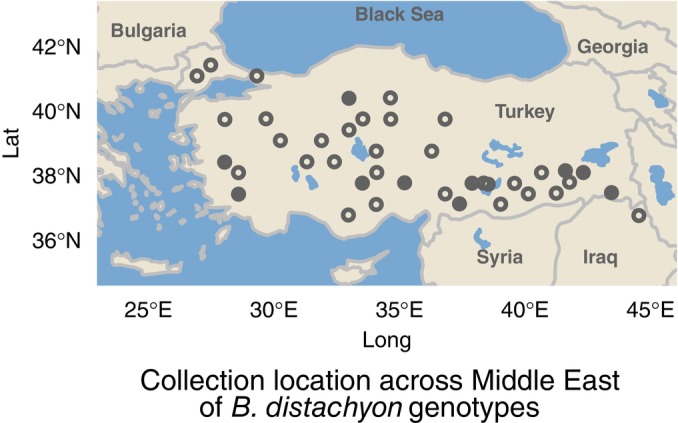
Overview of the distribution of *Brachypodium distachyon* genotypes used in our work herein. Plant germplasm was sourced from the USDA Germplasm Resources Information Network (GRIN) repository and was originally collected throughout Turkey, representing a portion of the *B. distachyon* native range. All points represent plant genotypes used in the first experiment in characterizing plant microbiomes. Closed points here represent genotypes that were additionally used in our second experiment evaluating plant responses to nitrogen.

To investigate the evolution of the plant‐associated microbiome composition and recruitment, we first characterized rhizosphere microbiomes across a panel of *Brachypodium distachyon* (L.) genotypes grown in a common glasshouse environment. *Brachypodium distachyon* is native to the Mediterranean region and found in dry, open habitats with well‐draining soils (Catalán *et al*., 2016). *Brachypodium distachyon* is a convenient model organism due to its small size, significant germplasm resources, and fast lifecycle, while also being closely related to many agronomically important grasses in the family *Poaceae* (Scholthof *et al*., 2018). We sourced *B. distachyon* germplasm from the USDA‐ARS Germplasm Resources Information Network (GRIN). We only considered diploid genotypes, as ploidy can influence microbial interactions (Forrester *et al*., 2020), as well as genotypes from a prior germplasm panel sourced from across Turkey, which includes inbred lines with associated genomic characterization using microsatellites (Simple Sequence Repeats, or SSR; Vogel *et al*., 2009). While not covering the full species distribution, we selected genotypes representing a diversity of environments by estimating historical environmental properties for each genotype's native habitat using publicly available datasets. This included SoilGrids, which predicts edaphic variables, including nitrogen content, cation exchange capacity (CEC), and pH, as well as a dataset generated by He *et al*. (2021) to predict available phosphorus (He *et al*., 2021; Poggio *et al*., 2021). We used WorldClim to predict climate variables, including average annual temperature and precipitation (Fick & Hijmans, 2017). Additionally, we extracted elevation from the metadata associated with each genotype in GRIN. We then chose 40 genotypes with a relatively even geographic spread across these variables (Figs 2, S1; Table S1).

#### Glasshouse experimental setup

We collected field soil from a prairie restoration site (40.128577, −88.140734) for use as microbial inoculum for our glasshouse experiment. While using soils from all 40 historic habitats would be ideal, as these would host microbiomes with which these genotypes evolved, this approach was not feasible, requiring distant sourcing and a large factorial glasshouse experiment. A single local inoculum was therefore necessary for feasibility. Moreover, any bias in using non‐native inoculum persists across all genotypes. We view this as a conservative approach in evaluating adaptive recruitment. We collected soil from approximately the first 50 cm of soil 1 wk before starting our experiment, storing samples at room temperature on the benchtop until use. We processed this inoculum through a 2‐mm soil sieve to remove large debris and plant matter. Then, we mixed the sieved soil with autoclaved ‘general‐purpose mix’: two parts peat, two parts perlite, and one part field‐collected soil produced by the UIUC Plant Care Facility (https://pcf.aces.illinois.edu/soil‐mixes/). We mixed the live inoculum with this glasshouse soil, with the inoculum making up 30% of the total volume, in a sterilized compost mixer, after which we used it to fill autoclave‐sterilized ‘cone‐tainer’ pots.

We prepared five replicate pots for each plant accession for a total of 200 pots (40 accessions × 5 replicates = 200). To remove seed‐surface contaminants, we sterilized the seed exteriors by vortexing seeds in a 3% sodium hypochlorite and 0.01% Tween‐20 solution for 2 min, followed by a thorough rinsing in sterile water. In each pot, we placed two to three seeds of the designated genotype and then covered the seeds with a thin layer of soil. Pots were arranged across five racks, representing five blocks, with one pot of each accession represented and randomly placed within each block. As plants began to emerge, we thinned pots to one plant per pot. Plants were grown in the glasshouse on a 26°C : 24°C, day : night schedule, supplemented with 14 h of daily light, for a total of 12 wk.

Following this growth period, we harvested samples by gently removing whole plants from their pots and clipping aboveground tissue. Similar to previous work (Burnham *et al*., 2022), we collected rhizosphere soils by first gently shaking the root system to remove general bulk soil, leaving solely the rhizosphere soil that immediately surrounded the plant roots. The remaining attached soil was collected in individual plastic bags for each plant, manually homogenized, and a subset was collected to fill 15‐ml tubes and stored at −20°C until further processing.

#### Sequencing preparation and processing

For DNA extraction, we first freeze‐dried the frozen soils and then transferred 0.25 g (±0.05) of soil per sample into 2‐ml Qiagen PowerPro Bead Tubes (Qiagen, Hilden, Germany). We then used the Qiagen PowerSoil Pro QIACube HT protocol using the QIACube HT extraction robot, following the manufacturer's protocol, except for the use of sterile 2‐ml microcentrifuge tubes instead of the Qiagen‐provided tubes. DNA concentrations were quantified using the Qubit 4 Fluorometer (Invitrogen), with the HS dsDNA Quantification Assay Kit (Invitrogen), and the purity of each sample (260 nm : 280 nm) was assessed using the NanoDrop One Spectrophotometer (ThermoScientific, Waltham, MA, USA). Samples were submitted to the Roy J. Carver Biotechnology Center at the University of Illinois for amplicon sequencing.

We used the Fluidigm Access Array IFC chip for the library preparation of our samples, as this sequencing approach allows for the simultaneous amplification of multiple amplicons (Favela *et al*., 2021). On the Fluidigm chip, we included universal primers for both bacterial (*16S* rRNA region) and fungal (*ITS2* region) amplicons. We additionally included primers targeting microbial functional groups associated with nutrient cycling, which are valuable in evaluating adaptive variation in the microbiome concerning the nutrient environment. We targeted nitrogen‐fixing bacteria, also known as diazotrophs (*nifH* gene), as this group influences the plant‐available nitrogen in the form of NH_4_^+^ (Smercina *et al*., 2019). We targeted arbuscular mycorrhizal fungi (AMF; *18S* rRNA gene), which can facilitate the uptake of mineral nutrients and water, and have evolutionary significance in the emergence of terrestrial plants (Feijen *et al*., 2018). Phosphorus‐provisioning taxa were targeted using the alkaline phosphatase functional gene (*phoD*), which mineralizes phosphate groups from organic phosphorus sources (Ragot *et al*., 2015). We amplified these gene targets using a PCR protocol that we have previously described (Favela *et al*., 2021), after which samples were sequenced on a 250‐cycle Illumina Novaseq lane. Specific amplicon sequencing primers are reported in Table S2.

We processed sequence data output using qiime2 (Caporaso *et al*., 2010), with each primer pair processed separately. We followed the recommended qiime2 pipeline (Hall & Beiko, 2018), including removing and trimming low‐quality reads, removing primer regions, merging forward and reverse reads, using dada2‐denoise to pool reads into amplicon sequence variants (ASVs), and removing chimeras. We identified the taxonomy for ASVs using the RDP naive Bayesian classifier, comparing each amplicon dataset to a corresponding reference database: bacterial *16S* rRNA to Greengenes2 (McDonald *et al*., 2024), fungal *ITS2* to UNITE (Abarenkov *et al*., 2010), mycorrhizal fungi *18S* to Maarj*AM* (Öpik *et al*., 2010), and nitrogen‐fixing *nifH* to ARB (Gaby & Buckley, 2014). We constructed a custom *phoD* database, pulling data from known alkaline phosphatase genes on the European Nucleotide Archive. In all, this pipeline produced high depth for each primer set (Table S3).

While our primers targeting the *nifH* gene capture microbial functions associated with nitrogen fixation, this only represents one component of the portions of the nitrogen cycle that influence plant‐available nitrogen. Ammonia oxidation, the microbial transformation of NH_4_^+^ to NO_3_^−^, may similarly be an important plant–microbial association as NO_3_^−^ is mobile and easily lost from the system via leaching (Cameron *et al*., 2013). Ammonia oxidation is highly phylogenetically constrained in both bacteria and archaea (Alves *et al*., 2018). Consequently, we created a new ASV table representing ammonia‐oxidizing microbes by directly subsetting both bacterial and archaeal lineages known to belong to this group from the *16S* rRNA ASV table (as done in Raglin *et al*., 2022). This ASV table was analyzed similarly to our other primer sets.

#### Statistical analysis

For each primer set, we chose a minimum target sequencing depth to ensure saturation in the observed ASV number (Table S3; Fig. S2). Samples below this depth were discarded, and the remaining samples were rarefied to these target depths to ensure even coverage. In all subsequent statistical analyses, we analyzed each amplicon dataset separately.

We first broadly evaluated the contribution of plant genotype to the composition of rhizosphere microbial communities using permutational analysis of variance models (PERMANOVA; Anderson, 2001). As predictors, we used plant genotype and glasshouse block. Given a variety of methods for generating microbiome distance matrices (Kers & Saccenti, 2022), we built separate models using the two most common and accepted distance metrics across the microbiome literature, Bray–Curtis and weighted‐Unifrac distance (Armstrong *et al*., 2022). Models were constructed in the R statistical environment using the vegan package (Oksanen *et al*., 2012). The primary distinction between these metrics is in Unifrac's incorporation of phylogenetic information, clustering communities together that are composed of different members but of the same phylogenetic clade. This phylogenetic approach can be powerful in large and diverse communities where phylogeny relates to function (Shankar *et al*., 2017). As our primers span phylogenetic scales, we elected to use both in our analyses.

We evaluated potential associations between rhizosphere microbiome structure and the abiotic characteristics of their historical environments. For each microbial group, we built distance‐based redundancy analysis (dbRDA) models using the dbrda function in the vegan R package. We correlated community structure with the environmental variables described above in our genotypic characterization, adding finer scale classifications for the precipitation: spring, summer, and fall precipitation, to account for seasonal water availability across the *B. distachyon* growing season in its historical environment (Fig. S3). We constructed separate models for both weighted‐Unifrac and Bray–Curtis distance matrices and, following similar methods as others (Singavarapu *et al*., 2023), used forward model selection with the ordistep function in the *vegan* package to identify variables that best explained variation in the microbiome. We included plant genotype and their spatial distribution as cofactors in these models. To this end, we first generated a distance matrix representing geographic distances between each genotype's historic habitat. We then generated a distance matrix representing genetic similarity among genotypes by calculating Hamming distances using SSR markers characterized on these genotypes (Vogel *et al*., 2009). We decomposed both of these matrices into a set of eigenvectors, from a Moran's eigenvector map, using the dbmem function in the adespatial package (Dray, 2016). These terms were incorporated using the Condition wrapper within the dbrda function, which partials out variation before fitting the terms of interest, allowing us to control for spatial and genomic autocorrelation before terms are run through model selection (Legendre & Legendre, 2012). For each model generated through model selection, we estimated the variance explained for each term by piping the model through the adonis2 function in vegan.

While these analyses can identify correlations between microbiomes and environmental variables, they do not reveal the nature of this association. We consequently included additional analyses to identify microbial lineages associated with significant environmental variables by building ANCOM‐BC models (analysis of compositions of microbiomes with bias correction) using the ANCOMBC R package (Lin & Peddada, 2020). We merged ASVs from identical phylogenetic bins into a single group for analysis. This approach can be interpreted as identifying how lineages respond instead of individual taxa, which allows for increased interpretability due to the large number of ASVs. As bacterial *16S* and fungal *ITS2* datasets represented broad phylogenetic surveys of these groups, we merged ASVs at the level of taxonomic order. As the remaining functional groups represented much narrower taxonomic groups, we merged ASVs at the level of genus. We included glasshouse block and genotype as random effects in these models. We followed the recommended pipeline, including sensitivity analyses and false discovery rate corrections.

Additionally, we assessed the correlation between these environmental variables and microbiome diversity metrics, as well as their relative abundance. For each sample, we estimated the richness and Pielou's evenness, common measures of microbiome diversity (Smith & Wilson, 1996; Kers & Saccenti, 2022). We also estimated the within‐genotype variation of the microbiome by calculating the distance to centroid (β‐dispersion) among the replicates of the same genotype (Anderson *et al*., 2006). All metrics were calculated using the *vegan* R package. And while we've focused on nutrient‐cycling functional groups by using targeted primers, some of these groups could additionally be subset out of the *16S* and *ITS2* datasets (as described with the ammonia oxidizers above). Although this approach relies on inference rather than direct functional gene quantification, it allows for the estimation of the relative abundance of these groups and assessment of their environmental drivers. We subset AMF from *ITS2* amplicons using ASVs classified to the Glomeromycota phylum. This primer has been shown to well capture Glomeromycota, though with some increased amplification bias (Bellemain *et al*., 2010). While all primer pairs may have biases toward or against specific groups, these biases are present across the whole dataset. We subset nitrogen‐fixing bacteria from the *16S* rRNA amplicons using ASVs that matched the identity of the ARB database we used for classification of *nifH* amplicons. We excluded the *phoD‐*harboring bacterial group from this analysis, as this group was not phylogenetically conserved, nor was our reference database highly specific. We assessed the environmental variables' contribution to microbiome traits by building mixed effects models, with glasshouse block and genotype as random effects. These models were built using the lme4 and lmertest R packages (Bates *et al*., 2015; Kuznetsova *et al*., 2015).

### Contributions of plant–microbiome interactions to the plant nitrogen response

#### Glasshouse experimental setup

While the above sequencing analysis sought to identify signatures of selection on plant–microbiome recruitment, we conducted a complementary glasshouse experiment to evaluate how microbiome variation influences the plant's response to its contemporary nutrient environment. We focused on how these communities contributed to the plant nitrogen response, as two of the microbial functional groups evaluated are core components of terrestrial nitrogen cycling. This second experiment could evaluate the impact of the associated plant microbiome on plant growth and, therefore, contribute to our understanding of whether these microbiome phenotypes represent adaptive variation. *A priori*, in a given nitrogen environment we expect plants to be locally adapted, with maximal growth from plant genotypes whose native habitat most resembles the current nitrogen environment. However, if variation in the recruitment of the nitrogen‐cycling microbes represents adaptive variation for their native nitrogen habitats, then adaptive nitrogen responses should be strongest only when grown with an available microbial community (Fig. 1c).

We evaluated *B. distachyon* nitrogen response in a second glasshouse experiment, using a subset of 10 genotypes distributed across the nitrogen gradient in the plants' original range. We grew plants in the glasshouse under either nitrogen‐limiting or sufficient conditions, while also crossing these treatments with either live or sterile soils to evaluate the microbial contribution to nitrogen adaptation. Briefly, we collected soil inoculum to provide microbes for plants from a restored prairie (same site and protocols as the above experiment). Sieved soil was mixed with steam‐sterilized glasshouse general‐purpose soil mix, with inoculum comprising 30% of the total volume. Half of this soil was then sterilized by autoclaving for 3 one‐hour cycles, with 30‐min rests between each cycle. These sterilized soils were used for the ‘sterile’ microbe treatment. Autoclave‐sterilized pots were filled with either ‘live’ or ‘sterile’ soils: 10 replicates per treatment combination for a total of 400 pots (10 plant genotypes × 2 nitrogen treatments × 2 microbe treatments × 10 replicates). Seeds from the selected genotypes were prepared for planting as previously described and arranged in the glasshouse in a randomized block design. Pots were watered every other day throughout the experiment.

As the soil was composed of 40% perlite, which contains no nutrients, and 40% peat, which has low‐nitrogen availability, these soils represented low‐nitrogen conditions. Plants assigned to nitrogen‐limited treatments received no added fertilizers, while nitrogen‐sufficient treatments received a pulse of nitrogen weekly, in the form of 4.3 mg of ammonium nitrate per pot. This fertilization level led to an addition of 12 g N m^−2^ over the course of the whole experiment. We chose this concentration as we aimed for a fertilization level similar to agronomic settings (Cao *et al*., 2018) to achieve maximal plant growth and represent conditions where nitrogen was not limited. While these two treatments do not precisely represent the native nitrogen conditions of *B. distachyon*, they capture extremes of nitrogen availability and were intended to test plant responses to either nitrogen limitation or nitrogen abundance.

We grew these plants in the glasshouse for 8 wk. As plants germinated, we thinned each pot to only contain a single seedling. A portion of pots did not have any successful germination (*c*. 25% of all pots, spread relatively evenly across treatments). We excluded these failed plants from all subsequent analyses. This left a total of 291 plants, approximately seven replicates per treatment combination. At the conclusion of this experiment, we harvested plants by gently removing them from their pot and clipping aboveground tissue from the root systems. We measured plant height, as well as whether they had begun tillering, an important stage in grass reproduction. Aboveground biomass was measured from oven‐dried tissues. While measuring reproductive fitness would have been ideal, *B. distachyon* genotypes have disparate flowering and seed set phenology (Schwartz *et al*., 2010), making such comparisons difficult in glasshouse settings. We note that while our measured traits are likely components of plant fitness (Mason *et al*., 2017; Younginger *et al*., 2017), they do not completely capture fitness, limiting our scope. We evaluated these proxy measures to characterize potential adaptive plant responses to their nitrogen environment.

#### Statistical analysis

We evaluated the plant nitrogen response by building mixed effects models for each trait, using the lme4 package. We included the following as fixed effects: a plant's historical nitrogen environment, glasshouse nitrogen environment (nitrogen‐limited or sufficient), microbe inoculum treatment (live or sterile soil), and the interaction of these terms. To control for nonindependence among individuals from the same genotype, we additionally included genotype as a random effect (Kawecki & Ebert, 2004), as well as the glasshouse block. As both height and biomass are continuous variables, we used normal distributions for these models, while tillering status as a binary variable required a binomial distribution.

Local adaptation is often evaluated in reciprocal transplant experiments by examining the two‐way interaction between an organism's source habitat and the contemporary habitat; in this case, the plant's source nitrogen habitat and the glasshouse nitrogen treatment (Blanquart *et al*., 2013). To determine whether plant–microbe interactions contribute to the plant nitrogen response, we focused on interaction terms involving the microbial treatment for each plant trait, as these terms may indicate the role of microbes in driving adaptation (as done by others, Ricks *et al*., 2023). An adaptive nitrogen response would be supported if we observed larger (biomass and height) plants when their current glasshouse nitrogen matches their historical environment (e.g. plants from low‐nitrogen habitats outperform others in the nitrogen‐limited treatment, plants from high‐nitrogen habitats are the best performers in the nitrogen‐sufficient treatment, Fig. 1c). We then evaluated the importance of microbes to this response by comparing plant trait responses across microbial treatments. Models including biomass as the explanatory variable produced singular warnings, as the genotype term could explain no additional variation. While this term could be dropped from the model, we chose to include it to correctly account for study design as replicates within a genotype represent pseudoreplication.

We further investigated the sign and direction of the microbial impact on plant height and biomass with a derived variable that quantified the difference between inoculated and sterile treatments, which we termed the ‘microbial effect’. We calculated the microbial effect for each plant trait by dividing the difference between live and sterile plants by the sterile plant trait value, an approach common in the plant–soil feedback literature ($\text{Microbial Effect}=\frac{\text{Live}-\text{Sterile}}{\text{Sterile}}$ Brinkman *et al*., 2010). Microbial effect > 0 indicate the live microbial inoculum increased the plant trait, and microbial effect is < 0 indicate the live microbial inoculum decreased the plant trait. These were calculated with mean values for each genotype, with the goal to relate microbial estimates with the historical nitrogen environment from which the plant genotypes were sourced. We characterized potential signatures of adaptation in these microbial effects by correlating these terms with their historic nitrogen environment. As adaptive nitrogen responses would be supported with differing reaction norms between the two nitrogen environments, we used a simulation approach to statistically compare the slopes of these reaction norms. Briefly, we first estimated uncertainty of the microbial effect by propagating error from the Live and Sterile estimates using the propagate R package (Spiess, 2013). With these we simulated data to estimate uncertainty on the slope coefficients, allowing us to derive *P*‐values in comparing the differences in slopes between nitrogen environments (for greater detail, see Methods S1).

Given the potential significant roles of nitrogen‐fixing and ammonia‐oxidizing bacteria in mediating nitrogen adaptation, we evaluated whether genotype‐level variation in these microbial groups could explain differences in the measured plant traits. This builds on prior studies demonstrating that the microbiome can be a genotype‐associated heritable trait (Peiffer *et al*., 2013; Deng *et al*., 2021; Boyle *et al*., 2024). Consequently, we treated microbiome characteristics measured as conserved traits of the plant genotypes. Specifically, we extracted elements of nitrogen cycler community structure, including the relative abundance of ammonia‐oxidizing bacteria and richness of nitrogen‐fixing bacteria observed for each plant genotype. These variables were used as predictors for the microbial impact on plant height and biomass. These microbiome traits were selected because they were significantly associated with the plant's historic nitrogen habitats (see the Results section). To evaluate the contribution of these elements of nitrogen cycler community structure to microbial benefits across the two nitrogen environments, we built similar models to those described above, by replacing the historic nitrogen environment with these microbiome variables. We note that this approach in using microbes as a conserved trait is limited, as there is significant plasticity in these components of the community between experiments, reducing the power of these analyses. Regardless, these analyses can provide valuable insights into the adaptive benefits of microbial functional groups.

## Results

### Linking plant–microbiome structure with their historical environments

**Fig. 3:**
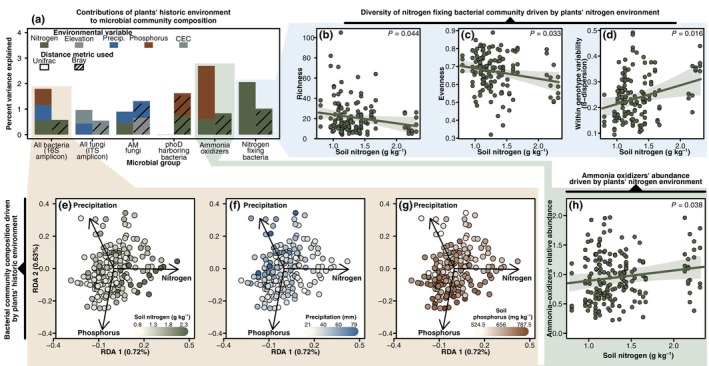
Displaying significant drivers of *Brachypodium distachyon* microbiome structure, specifically the plants' historical environment as predictor variables. (a) Visual summation of distance‐based redundancy analysis (dbRDA) models built for each microbial group. Stacked bars total to the amount of variation that can be explained for each microbial functional group using the historical environment, partitioning the size of bars to the amount explained by each variable. Only variables chosen through forward model selection were included. Separate models were constructed using weighted‐Unifrac and Bray–Curtis dissimilarities for each microbial group and are displayed with open vs cross‐hatched bars, respectively. To reduce complexity, all precipitation variables were combined together here into one color. As the historical soil nitrogen was a significant driver of the nitrogen‐fixing community, the blue inset (b–d) displays how nitrogen contributes to the various characteristics of the diversity of this group. This features the correlation between historical soil nitrogen and total richness, evenness, and the β‐dispersion. Lines represent the estimated model correlating these variables, with their corresponding prediction interval. *P*‐values, displayed in the top corner, are the output of these models. As historical soil nitrogen, soil phosphorus, and precipitation were significant drivers of the bacterial community (*16S rRNA* gene, V4 amplicon), the yellow inset displays their impact on composition. Constrained ordinations (dbRDA) to fit these three variables and displayed three times (e–g), with points colored accordingly to the three variables. Finally, as soil nitrogen was a significant driver of the ammonia‐oxidizing microbial community, the green inset (h) displays how nitrogen contributes to the relative abundance of this group.

Across all microbial groups, plant genotype was a significant driver of microbiome structure in at least one of the PERMANOVA models, either with Bray–Curtis or Unifrac distance, explaining 21% to 31% of the variation (Table 1). Previous reports of genotype effect size ranged from 3% to 32%, placing these results in a similar context (Cregger *et al*., 2018; Liu *et al*., 2019; Favela *et al*., 2021). Model selection of variables in the dbRDA identified a portion of this between‐genotype microbiome variation associated with the plants' historical environments, explaining between *c*. 1% and 3% of the variation (Fig. 3a; Tables S4, S5). For the bacterial community, model selection identified soil nitrogen, soil phosphorus, and precipitation as significant variables, explaining maximally *c*. 1.8% of the variation (Fig. 3e–g). For the fungal community, soil CEC and precipitation were identified as significant predictors, explaining *c*. 1% of the variation. In the AM fungal community, nitrogen, precipitation, and elevation were significant predictors, explaining *c*. 1.4% of the variation. Soil nitrogen and phosphorus significantly explained 1.7% of the variation in the community of bacteria harboring *phoD*. For the bacterial nitrogen‐fixers, 2% of the variation could be explained by soil nitrogen, while soil nitrogen and phosphorus could explain 3% of the variation in the community of ammonia oxidizers.

**Table 1:** PERMANOVA models for the effect of *Brachypodium distachyon* genotype and block on each microbial group examined herein.

Target group	Weighted‐Unifrac distance		Bray–Curtis distance	
	Genotype	Block	Genotype	Block
Bacteria	22.9%***	3.5%***	22.8%***	3.7%***
Fungi	21.6%*	2.7%***	21.8%**	2.8%***
AM fungi	23.2%**	2.8%*	24.5%***	3.2%***
*phoD*‐harboring bacteria	26.1%	3.1%	28.7%*	2.8%
N‐fixing bacteria	26.5%*	3.2%^+^	31.6%	2.5%
Ammonia‐oxidizing microbes	23.8%^+^	1.2%	23.2%***	2.5%^+^

We display models using either weighted‐Unifrac or Bray–Curtis distance, with the *F* value for each model term. Terms with a *P*‐value < 0.1 are indicated with the following symbology: ^+^, *P* ≤ 0.1; *, *P* ≤ 0.05; **, *P* ≤ 0.01; ***, *P* ≤ 0.001.

We identified a variety of microbial lineages whose relative abundance was significantly correlated with the host's historical environment (Figs S4–S7). Briefly, across all bacteria identified, soil nitrogen was correlated with 13 orders, precipitation with 10 orders, and soil phosphorus with 10 orders. Across all fungi, precipitation was correlated with two orders, and soil CEC was correlated with two orders. In the *phoD* community, soil nitrogen and phosphorus were each correlated with one genus. In the nitrogen‐fixing community, soil nitrogen was correlated with two genera. No nitrifier or AMF lineages were significantly correlated with their environmental predictors.

Largely, the historic environment was not correlated with any diversity metrics across the microbiome (Figs S8–S12). One of the exceptions included the diversity of nitrogen‐fixing bacteria, which was significantly correlated with the historic soil nitrogen (Fig. 3b–d). Community richness of the nitrogen‐fixers significantly decreased as plants originated from increasingly nitrogen‐rich habitats, moving from an average of 25 ASVs on genotypes from nitrogen‐poor habitats to 12 ASVs on genotypes from nitrogen‐rich habitats. A similar relationship was observed for nitrogen‐fixer evenness. Conversely, β‐dispersion increased as genotypes were sourced from increasingly nitrogen‐rich habitats.

In examining potential drivers of the relative abundance of the three functional groups, only ammonia‐oxidizing bacteria showed a significant relationship with historic nitrogen availability (Figs 3h, S13, S14). The relative abundance of the ammonia‐oxidizing community significantly increased as plants originated from increasingly nitrogen‐rich habitats, moving from an average of 0.85% of the community on genotypes from nitrogen‐poor habitats to 1.12% on genotypes from nitrogen‐rich habitats.

### Contributions of plant–microbiome interactions to the plant nitrogen response

**Fig. 4:**
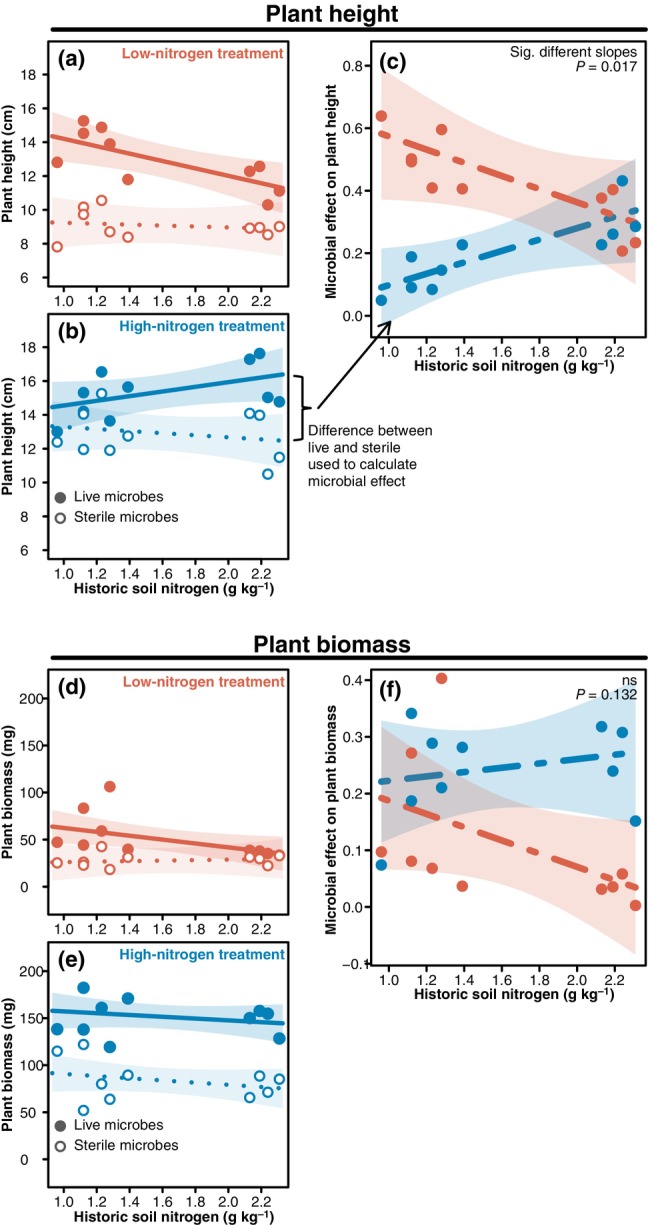
Response of plant traits to nitrogen, including plant height (a, b) and biomass (d, e). Data are split to display plant traits when grown in low‐nitrogen treatments (a, d) and high‐nitrogen treatments (b, e). Points represent the mean trait estimates for each of the *Brachypodium distachyon* genotypes and are split between when genotypes are grown using soils with live microbes (closed circles) vs with soils using sterile microbes (open circles). Lines in each panel represent the estimated model, with their corresponding prediction interval. As we are interested in how a plant's historic nitrogen habitat mediates plant nitrogen responses, these plant traits are correlated with each genotype's historic soil nitrogen level. We additionally display the estimated effect of microbes on each genotype's trait (c, f). Microbial impact is calculated as the response ratio of the plant trait from plants provided with live inoculum vs plants provided with sterile inoculum. We note that each of these points has an associated error, which we do not display here for clarity. We display in the top right corner our confidence in whether there is a difference in the slopes between the two environments in the form of a *P*‐value. *P*‐values were derived from a simulation approach described in the text.

In evaluating microbial contributions to the plant nitrogen response, our plant traits (biomass, height, and tillering) were significantly influenced by nitrogen addition and microbial treatment (Table 2). Both nitrogen and the addition of microbes increased the size of the plant across all plant traits; however, for biomass and height, these variables significantly interacted (Fig. 4a,b,d,e). Additionally, for height, these variables interacted with their historic nitrogen history.

**Table 2:** ANOVA tables from mixed effect models, assessing the drivers of *Brachypodium distachyon* height, biomass, and tillering status.

Variable	df	Biomass	Height	Tillering
Historic Nitrogen	1	3.87^+^	0.71	0.78
Microbe	1	36.48***	39.91***	6.26***
Glasshouse Nitrogen	1	208.97***	114.77***	22.61***
HistN : Microbe	1	1.10	0.01	0.83
HistN : GlasshouseN	1	< 0.00	9.72**	0.29
GlasshouseN : Microbe	1	16.03***	5.86*	0.84
HistN : GlasshouseN : Microbe	1	1.37	12.47***	0.09
Residuals	283			

Models included the following as fixed effects: the historic nitrogen environment from which plants were collected, the microbe status in glasshouse (live vs sterile soils), the nitrogen status in the glasshouse (high‐ vs low‐nitrogen treatment), and the interactions between these terms. Biomass and Height were evaluated using normal distributions, and values displayed are *F* statistics for each term. Tillering was evaluated using binomial distributions, and values displayed are χ^2^. Terms with a *P*‐value < 0.1 are indicated with the following symbology: ^+^, *P* ≤ 0.1; *, *P* ≤ 0.05; **, *P* ≤ 0.01; ***, *P* ≤ 0.001.

To parse these interactions, we examined the microbial effect (ratio between the live and sterile treatments) across genotypes. We found that the microbial effect on height was correlated with the plant's historical nitrogen environment, but the direction of this relationship depended on the glasshouse nitrogen treatment (Fig. 4c, *P* = 0.024). For example, the microbial effect on plant height in the low‐nitrogen treatments decreased for genotypes sourced from higher nitrogen environments, but in high‐nitrogen treatments, increased for genotypes sourced from higher nitrogen environments. While results trended in the same direction for the microbial effect on biomass, these were nonsignificant (Fig. 4f, *P* = 0.132).

**Fig. 5:**
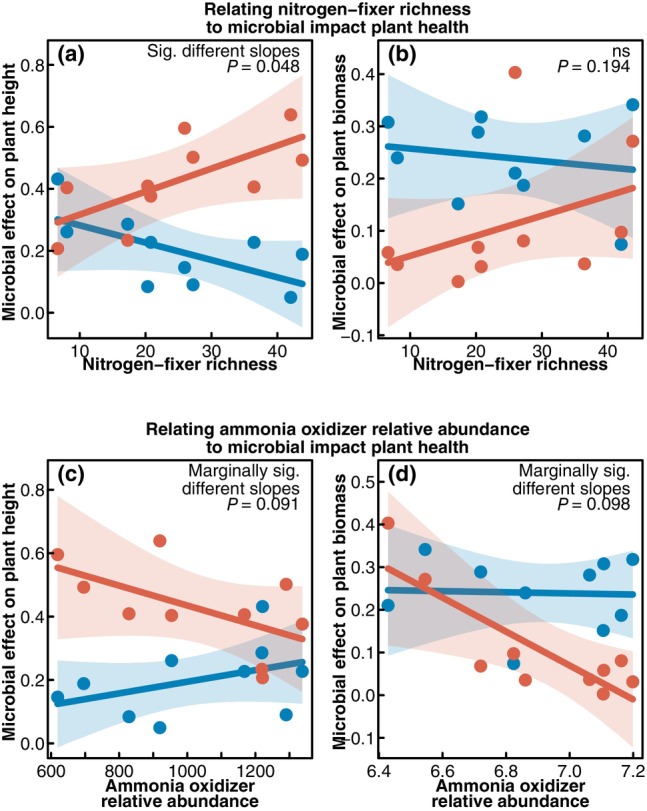
Correlation between nitrogen‐cycling microbiome composition for each genotype of *Brachypodium distachyon* and the associated microbial effect on plant biomass and height. These represent nitrogen‐fixer richness (a, b) and ammonia‐oxidizing microbial relative abundance (c, d). The microbial effect terms are the same as those represented in Fig. 4 but recoded with new *x*‐axis terms. The microbial effects from low and high‐nitrogen glasshouse treatments are differentiated with orange vs blue coloring, respectively. Lines in each panel represent the estimated model, with their corresponding prediction interval. We display in the top right corner our confidence in whether there is a difference in the slopes between the two environments, in the form of a *P*‐value. *P*‐values were derived from a simulation approach described in the text. The live and sterile components of these can be seen in Supporting Information Fig. S15.

The plant nitrogen response was also significantly associated with the structure of their associated nitrogen‐cycling community. Both ammonia‐oxidizing microbial relative abundance and nitrogen‐fixing bacterial richness were correlated with plant height and biomass (Tables S6, S7; Figs 5, S15), although these effects depended on both the nitrogen addition and the presence of a live soil inoculum. As above, we explored these interactions by correlating the nitrogen‐cycling community structure with the microbial effect. Nitrogen‐fixer richness was associated with increased microbial benefits to plant height under low‐nitrogen conditions, while this response was negative under high‐nitrogen conditions (*P* = 0.048). While trending in a similar direction, these results were not significant for the microbial impact on biomass (*P* = 0.194). Similarly, ammonia oxidizing relative abundance was associated with decreased microbial benefit to both height and biomass under low‐nitrogen conditions, while this response shifted to be positive or neutral under high‐nitrogen conditions (marginal effects, Height: *P* = 0.091, Biomass: *P* = 0.098).

## Discussion

Prior work has highlighted that host genotype is a strong driver of its associated microbiome composition (Cregger *et al*., 2018; Liu *et al*., 2019; Favela *et al*., 2021), with genotypic differences attributed to specific heritable host traits and genetic elements that influence microbiome assembly (Wallace *et al*., 2018; Deng *et al*., 2021; Van Wallendael *et al*., 2022; Boyle *et al*., 2024). Our results here are consistent with such work, with *Brachypodium distachyon* genotype a significant predictor in the composition of its rhizosphere microbiome. Importantly, we found a portion of this between‐genotype microbiome variation was explained by the historical climate and edaphic environment of these genotypes, including both broad compositional patterns in the microbiome and the abundance of specific microbial lineages. While this between‐genotype microbiome variation explained by these was relatively small, small effect sizes are common in microbiome studies (e.g. see Barnard *et al*., 2013). Moreover, these effects may be underestimates due to limitations in environmental data resolution and the exclusion of potentially relevant variables, such as additional edaphic factors and ecological interactions with soil microorganisms from *B. distachyon's* native range. Regardless, because all genotypes were grown in a common glasshouse environment with a standard soil microbial inoculum, these results suggest that various heritable plant traits driving microbiome recruitment may be under selection by the abiotic environment, producing the signature of a plant's historical environment in its associated microbiome when grown elsewhere. As nitrogen‐cycling microbes play a critical role in plant adaptation to nitrogen‐limited environments (Martin *et al*., 2017), we use this group to explore below if this genotypic variation is shaped by adaptive recruitment. Adaptive recruitment would be supported when genotypes from a given nitrogen habitat are colonized by locally beneficial nitrogen cyclers (Fig. 1b), with plant adaptation in these environments driven by the presence of these microbes (Fig. 1c).

Our sequencing of the diazotroph community supports our hypothesis of adaptive recruitment, as the community was ideally structured to benefit *B. distachyon* genotypes in their historical nitrogen environment. Plants from low‐nitrogen habitats were colonized by a greater diversity of nitrogen‐fixing bacteria, which may maximize nitrogen provisioning in their historic nutrient‐limited habitats. While nitrogen fixation is typically associated with specialized bacterial symbioses, such as the legume–rhizobia system, associative nitrogen‐fixing bacteria are commonly found to colonize the carbon‐rich rhizosphere zones of grasses (Smercina *et al*., 2019). Grasses can influence free‐living diazotroph recruitment through the release of specific mucilage from their roots (Bennett *et al*., 2020). For example, a maize landrace with extensive aerial roots secretes a sugar‐rich mucilage that supports highly diverse and active nitrogen‐fixing communities to supply the plant's nitrogen needs (Van Deynze *et al*., 2018). Similarly, the interior tissues of early successional grass species can be enriched with nitrogen‐fixing microbes supporting their growth in the nutrient‐poor conditions of newly exposed glacial tills (Sun *et al*., 2022). While the specific traits mediating the recruitment of diazotrophs in *B. distachyon* are unknown, our results support the hypothesis that low‐nitrogen environments may have selected for *B. distachyon* root traits that promote diazotroph recruitment. This hypothesis is consistent with our measures of low evenness and high β‐dispersion in the nitrogen‐fixing communities. β‐dispersion is a measure of microbiome variance and can indicate the relative strength of stochastic assembly in the microbiome, as such processes will increase the between‐replicate diversity within the same treatment (Stothart *et al*., 2021). And indeed, genotypes native to high‐nitrogen habitats hosted nitrogen‐fixing bacterial communities more likely to be dominated by only a few taxa, with overall increased β‐dispersion in the community. These microbiome characteristics may indicate relaxed selection on plant traits controlling the recruitment of diazotrophs in high‐nitrogen habitats, allowing random and potentially less effective nitrogen‐fixers to colonize. By contrast, genotypes native to low‐nitrogen environments maintain more diverse diazotroph assemblages with lower variance, potentially indicating selection for efficient recruitment in this functional group for plants originating from nitrogen‐limiting conditions.

Ammonia‐oxidizing microbial communities showed similar patterns, reflecting the influence of the plant's historical nitrogen environment on community composition. Ammonia‐oxidizing microbes transform ammonium – an immobile, plant‐available form of nitrogen – into nitrate, a form that is more mobile and easily lost via leaching (Cameron *et al*., 2013). The activity of these microbes can reduce nitrogen availability and thus negatively influence plant fitness. Adaptive variation in these plant communities may be represented by reduced activities of ammonia oxidizers in low‐nitrogen habitats, as their presence would disproportionately harm plant fitness through competition or loss of nitrogen resources. Consistent with this prediction, the relative abundance of ammonia oxidizers was significantly lower in genotypes originating from nitrogen‐deficient habitats. Several grass species can suppress ammonia oxidizers through root‐derived compounds known as biological nitrification inhibitors (BNI; Kuppe & Postma, 2024). *Brachypodium distachyon* may similarly produce BNI compounds, and populations in nitrogen‐limited habitats may be under selective pressure to enhance this suppression as a means of conserving ammonium and ensuring access to bioavailable nitrogen resources. Future exploration and validation of potential BNI compounds in *B. distachyon*, as well as their distribution across the landscape, may yield insight into their evolutionary drivers.

Our experimental glasshouse work partially supports our hypothesis that genotypic differences in microbiome recruitment represent adaptive variation, as microbial interactions shaped the plant nitrogen response. For example, microbes provided the largest increases in plant height when plants were grown in nitrogen environments similar to their historic environment (this was marginal for biomass), indicating that microbial interactions play a potentially adaptive role in the nitrogen response, consistent with our hypotheses (Fig. 1c). We do note that neither of our measured plant traits is a direct measure of fitness (although others have used these traits as fitness proxies, Ludwig *et al*., 2024), making direct inferences to local adaptation difficult. Moreover, we cannot identify the specific microbes mediating this response, as our microbial treatments simply differed in the presence or absence of soil microbes. However, diazotrophs and ammonia oxidizers are likely contributors, given their role in mediating nitrogen availability. And indeed, the relative abundance of ammonia oxidizers and diazotroph richness were associated with the microbial impact on the plant, contingent on the nitrogen treatment. These context‐dependent effects indicate that variation in plant–microbiome recruitment may have measurable consequences for plant growth. Future studies using controlled microbial inoculations, linking direct measures of microbial recruitment to real plant fitness, could disentangle the contributions of these nitrogen‐cycling functional groups.

Overall, our results suggest plant recruitment of the nitrogen‐cycling community may be adaptively influenced by local selective pressures. Plant adaptation to low‐nitrogen environments has been previously observed (Macel *et al*., 2007), and while plant traits, such as increased nutrient scavenging, may be contributors here, our work may also point to the joint plant–microbe interaction. Low‐nitrogen habitats limit plant growth and may select for plant traits, such as the production of BNI exudates to suppress ammonia oxidizers or increased mucilage production to support nitrogen‐fixing bacteria, that can alleviate this stress. As the traits may carry energetic costs (Lata *et al*., 2022), they may present a tradeoff between low‐ and high‐nitrogen environments, where traits advantageous under nitrogen limitation are disfavored in high‐nitrogen settings. Future work may identify the specific plant traits underlying this adaptation.

While our work focused on nitrogen‐cycling taxa, this framework may apply to other microbial functional groups that vary across environmental gradients. For example, variation in AM fungal communities was significantly explained by their host plants' historic precipitation and nitrogen environment (Fig. 3a; Tables S4, S5), consistent with their role in enhancing plant drought tolerance (Bahadur *et al*., 2019) and nitrogen acquisition (Jin *et al*., 2012). Similarly, the structure of *phoD‐*harboring communities, which can influence phosphorus availability, was significantly correlated with the plant's historical phosphorus environment (Fig. 3a; Tables S4, S5), suggesting a role in plant phosphorus adaptation. In these patterns, we see the possibility that plants adapt both directly, through physiological traits, but also indirectly, through their influence on microbiome functions related to environmental adaptation. Future work may try to connect variation in these, as well as other potential groups, to patterns of plant adaptation. However, not all our results may necessarily be indicative of adaptive microbiome variation. For example, the *phoD‐*harboring community was additionally associated with the historical nitrogen environment; it is not immediately clear how this microbial function may play an adaptive role across a nitrogen gradient. This pattern, as well as others across the diversity of the bacterial and fungal lineages we observed responding to the historical abiotic environment, may reflect neutral variation or result from selection on unrelated plant traits that incidentally impact the microbiome. Indeed, as highlighted earlier, adaptive and neutral variation in microbiomes is not necessarily mutually exclusive, potentially dependent on the specific microbial group and environment. Combining evolutionary ecology, plant physiology, soil biogeochemistry, and microbial ecology may be necessary to disentangle the complex interactions contributing to plant adaptation.

Beyond their ecological functions, these findings have broader implications for the role microbes play in shaping plant evolution. While others have shown locally adaptive plant responses can depend on the presence of microbial partners (Petipas *et al*., 2020), we suggest here that such adaptation is driven through recruiting specific, locally beneficial microbial taxa. The joint abiotic and microbial selective pressures may be driving plants to optimize microbial recruitment and interactions. Traits that were previously considered as solely plant‐derived (e.g. nutrient uptake, disease resistance, or stress tolerance) may rather be the product of adaptive recruitment and filtering of the microbiome to achieve a specific phenotype (Favela *et al*., 2021). While the microbial role in adaptation has been well studied in specialized mutualisms, such as mycorrhizal fungi and the legume–rhizobia system (Martin *et al*., 2017), our work suggests that diffuse community‐level associations could underlie plant local adaptation. Indeed, as small changes in the microbiome can have outsized impacts on plant health (Márquez *et al*., 2007), even minimal changes to the plant gene networks underlying microbiome assembly may drive rapid evolutionary change. Although our study did not identify specific mechanisms by which plants may be adapting to alter microbiome recruitment, prior work has identified numerous potential traits, including root exudation, internal chemistry, or immune function (González‐Teuber *et al*., 2020; Eichmann *et al*., 2021; Seitz *et al*., 2022; Galindo‐Castañeda *et al*., 2024) as examples of small but significant changes. We see future studies addressing this area by combining large plant genomic and microbiome datasets to identify the genomic underpinnings of these interactions and how they evolve across the landscape.

These results also have important implications for understanding plant evolution in managed systems. Domestication and modern plant breeding have unintentionally disrupted beneficial plant–microbiome interactions (Barnes *et al*., 2024), leading to reduced recruitment of high‐quality mycorrhizal fungi and rhizobia (Kiers *et al*., 2007; Martín‐Robles *et al*., 2018), and growth‐promoting bacteria (Valente *et al*., 2020), and increased susceptibility to microbial pathogens (Nygren *et al*., 2015; Soltis *et al*., 2019). Recent work has shown that modern maize lineages recruit microbiomes with maladaptive nitrogen‐cycling assemblages (Favela *et al*., 2021). Modern maize (developed since the Green Revolution) recruits fewer nitrogen‐fixing taxa, along with a greater relative abundance of ammonia‐oxidizing and denitrifying bacteria, compared with wild and landrace maize. Our results highlight that these shifts may be driven by relaxed selection on plant traits that influence rhizosphere microbiome establishment, especially in agricultural systems with high‐nutrient inputs that remove any selective pressure to recruit nitrogen‐provisioning microbiomes (Meier *et al*., 2022). These insights provide some guiding principles for incorporating microbiome‐associated sustainability traits into crop breeding programs (Raglin & Kent, 2025).

Overall, this study suggests that the abiotic environment may act as a selective agent to shape plant genotypic variation in rhizosphere microbiome recruitment. Indeed, these microbial partnerships may be pathways to plant adaptations, with plants evolving to recruit optimal microbiomes for their local environments. As global change rapidly alters environmental stability, understanding the contribution of rhizosphere microbiomes to plant adaptation and how these interactions shift across environments is necessary for agricultural sustainability, ecosystem conservation, and restoration. Expanding this framework to include additional microbial functional groups and their roles in mediating adaptive or maladaptive plant phenotypes should be areas of future study.

## Competing interests

None declared.

## Author contributions

KDR and SSR conceived the initial design of the experiment and carried out the glasshouse experimental work. SSR performed the molecular work. KDR conducted the bioinformatics and statistical analyses with input from both SSR and ADK. KDR wrote the first draft of the manuscript. All authors contributed to review and editing.

## Disclaimer

The New Phytologist Foundation remains neutral with regard to jurisdictional claims in maps and in any institutional affiliations.

## Supporting information


**Dataset S1** The estimated historic environmental data for each plant genotype, derived from publicly available datasets, as well as the plant phenotypic data from our nitrogen manipulation experiment.

**Fig. S1** Distribution of estimated historic environmental variables for the *Brachypodium distachyon* genotypes used in our work.
**Fig. S2** Rarefaction curves for each amplicon sequenced.
**Fig. S3** Estimated precipitation of the native habitats of our *Brachypodium distachyon* genotypes.
**Fig. S4** Historical environments of *Brachypodium distachyon* influencing the enrichment of specific bacterial 16S lineages.
**Fig. S5** Historical environments of *Brachypodium distachyon* influencing the enrichment of specific fungal ITS lineages.
**Fig. S6** Historical environments of *Brachypodium distachyon* influencing the enrichment of specific phoD‐harboring bacterial lineages.
**Fig. S7** Historical environments of *Brachypodium distachyon* influencing the enrichment of specific nitrogen‐fixing bacterial lineages.
**Fig. S8** Historical environments of *Brachypodium distachyon* influencing the diversity of the bacterial 16S community.
**Fig. S9** Historical environments of *Brachypodium distachyon* genotypes influencing the diversity of the fungal ITS community.
**Fig. S10** Historical environments of *Brachypodium distachyon* genotypes influencing the diversity of the AM fungal community.
**Fig. S11** Historical environments of *Brachypodium distachyon* genotypes influencing the diversity of the phoD‐harboring bacterial community.
**Fig. S12** Historical environments of *Brachypodium distachyon* genotypes influencing the diversity of the ammonia‐oxidizing microbial community.
**Fig. S13** Correlation of nitrogen‐fixing bacterial relative abundance with soil nitrogen.
**Fig. S14** Correlation of AM fungal relative abundance with precipitation and elevation.
**Fig. S15** Correlation between nitrogen‐cycling microbiome composition for each genotype with their height and biomass.
**Methods S1** Outline for simulation approach to evaluate difference in plant reaction norms between the two nitrogen environments.
**Table S1** List of *Brachypodium distachyon* genotypes used in this work, including their USDA accession number and original collection location.
**Table S2** Primer sets used for the Fluidigm sequencing run.
**Table S3** Summary statistics for DNA sequencing.
**Table S4** Results from dbRDA models correlating community composition with weighted‐Unifrac distances.
**Table S5** Results from dbRDA models correlating community composition with Bray–Curtis distances.
**Table S6** ANOVA tables representing ammonia oxidizer abundance as a predictor for plant traits.
**Table S7** ANOVA tables representing nitrogen‐fixing richness as a predictor for plant traits.
Please note: Wiley is not responsible for the content or functionality of any Supporting Information supplied by the authors. Any queries (other than missing material) should be directed to the *New Phytologist* Central Office.

## Data Availability

All sequence data are available at the NCBI SRA database (accession no.: PRJNA1269615) (https://www.ncbi.nlm.nih.gov/bioproject/PRJNA1269615). Additional data have been attached as a supporting document (Dataset S1), which includes plant genotypes' historic environments, as well as the plant phenotypic data from the nitrogen response glasshouse experiment.
