# Supplementary material for: Signatures of local nitrogen adaptation in the Brachypodium distachyon root microbiome
Source: New Phytol. 2025 Oct 29;249(1):433–47. doi: 10.1111/nph.70684 (PMC12676078; doi:10.1111/nph.70684)
Supplement: Supplementary file 2 — Fig. S1 Distribution of estimated historic environmental variables for the Brachypodium distachyon genotypes used in our work. Fig. S2 Rarefaction curves for each amplicon sequenced. Fig. S3 Estimated precipitation of the native habitats of our Brachypodium distachyon genotypes. Fig. S4 Historical environments of Brachypodium distachyon influencing the enrichment of specific bacterial 16S lineages. Fig. S5 Historical environments of Brachypodium distachyon influencing the enrichment of specific fungal ITS lineages. Fig. S6 Historical environments of Brachypodium distachyon influencing the enrichment of specific phoD‐harboring bacterial lineages. Fig. S7 Historical environments of Brachypodium distachyon influencing the enrichment of specific nitrogen‐fixing bacterial lineages. Fig. S8 Historical environments of Brachypodium distachyon influencing the diversity of the bacterial 16S community. Fig. S9 Historical environments of Brachypodium distachyon genotypes influencing the diversity of the fungal ITS community. Fig. S10 Historical environments of Brachypodium distachyon genotypes influencing the diversity of the AM fungal community. Fig. S11 Historical environments of Brachypodium distachyon genotypes influencing the diversity of the phoD‐harboring bacterial community. Fig. S12 Historical environments of Brachypodium distachyon genotypes influencing the diversity of the ammonia‐oxidizing microbial community. Fig. S13 Correlation of nitrogen‐fixing bacterial relative abundance with soil nitrogen. Fig. S14 Correlation of AM fungal relative abundance with precipitation and elevation. Fig. S15 Correlation between nitrogen‐cycling microbiome composition for each genotype with their height and biomass. Methods S1 Outline for simulation approach to evaluate difference in plant reaction norms between the two nitrogen environments. Table S1 List of Brachypodium distachyon genotypes used in this work, including their USDA accession number and original collection location. T [file NPH-249-433-s001.docx]

## *New Phytologist* Supporting Information

Article title: Signatures of local nitrogen adaptation in the Brachypodium distachyon root-microbiome

Authors: Kevin D. Ricks, Sierra S. Raglin, & Angela D. Kent

Article acceptance date: 9 October 2025

The following Supporting Information is available for this article:

**Fig. S1**

Distribution of estimated historic environmental variables for the *Brachypodium distachyon* genotypes used in our work. Variables were estimated through various models, including WorldClim, SoilGrids, and a dataset generated in He *et al*. (2021). Details can be found in Table S1.


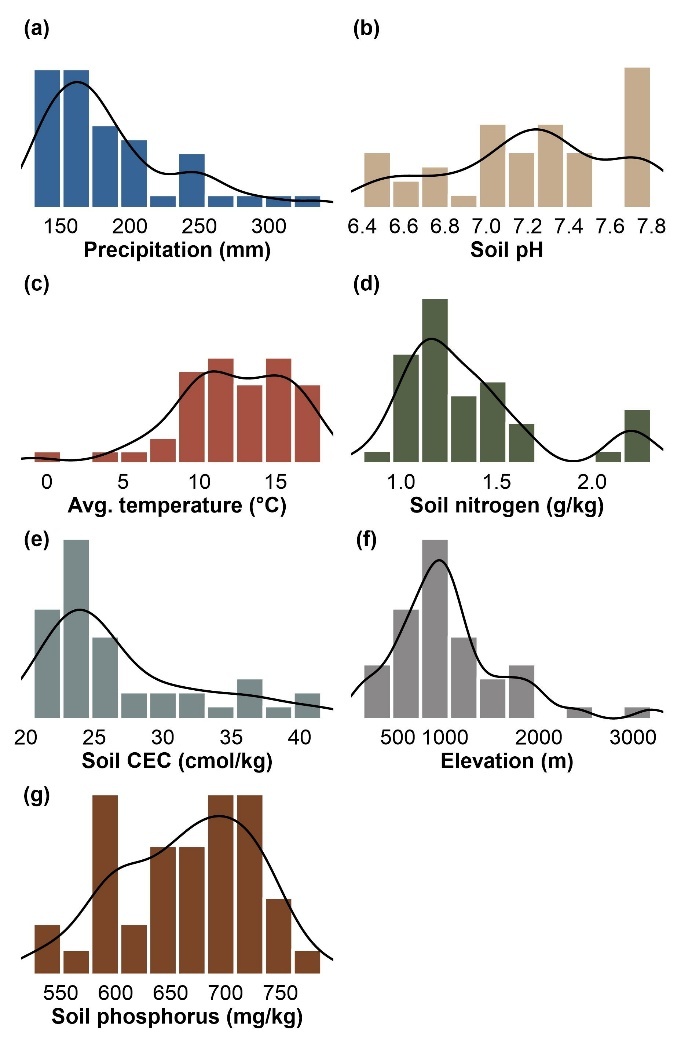


**Fig. S2**

Rarefaction curve for each sample across each microbial target group. The red dashed line represents the target rarefaction depth for each group. Ammonia oxidizers (both bacterial and archaeal) are not displayed here, as this group was pulled from the *16S rRNA* dataset.


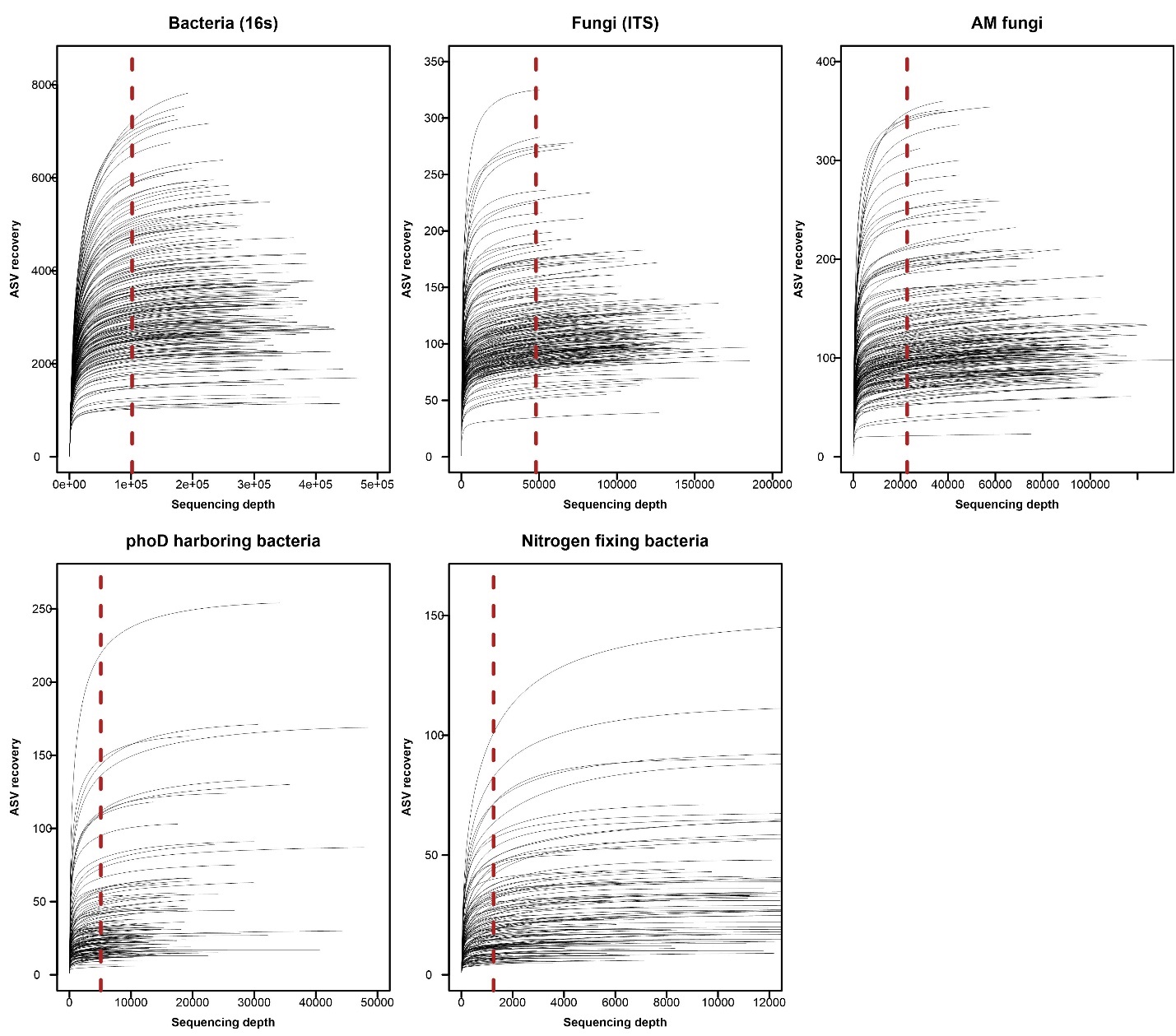


**Fig. S3**

Estimated precipitation of the native habitats of our 40 *Brachypodium distachyon* genotypes, broken down into the 3 potential growing seasons, Spring (March-May), Summer (June-August), and Fall (September to November). Each panel shows the correlation between these seasons.


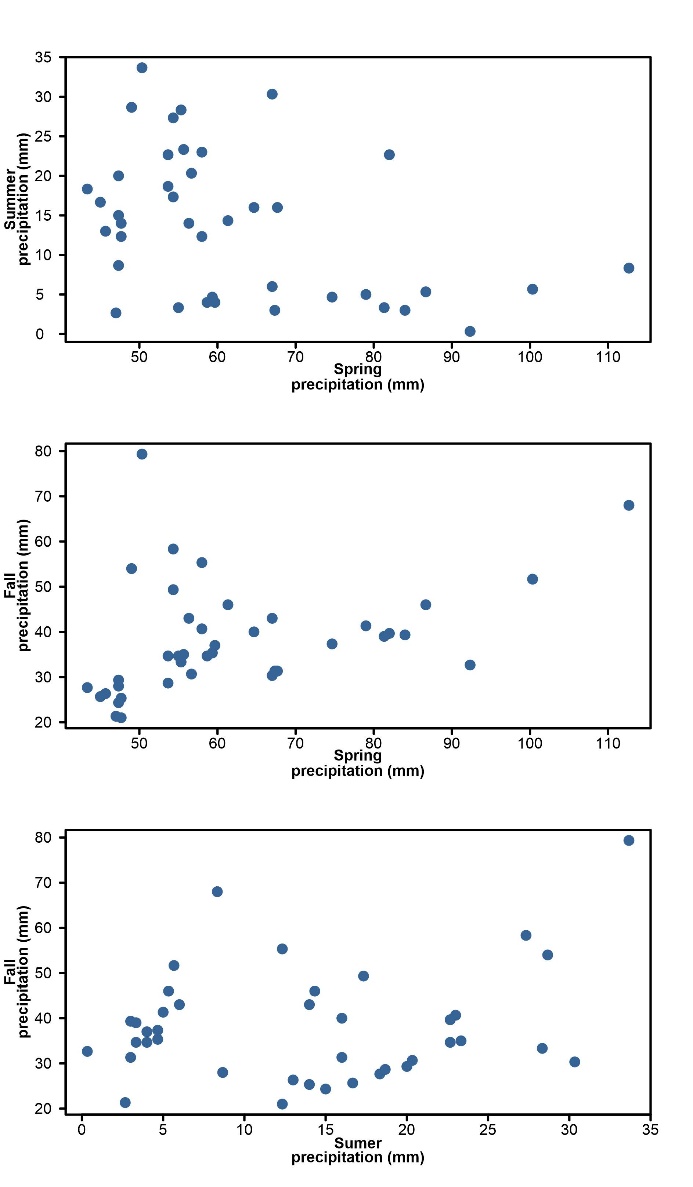


**Fig. S4**

Significant drivers of enrichment in bacterial 16S lineages across *Brachypodium distachyon* genotypes, specifically using the home environment from which plants were collected as predictors. We display the correlations between soil nitrogen, phosphorus, and precipitation with the enrichment of specific microbial lineages as variables were identified as significant in the dbRDA model. To reduce complexity, ASVs were grouped by the taxonomic order to which they were assigned, and evaluated the significance using ANCOMBC. Lineages with significant correlations are labeled on the phylogeny. The lineages are colored based on which variables were significant drivers, with closed vs open circles denoting whether these were positive vs. negative association.


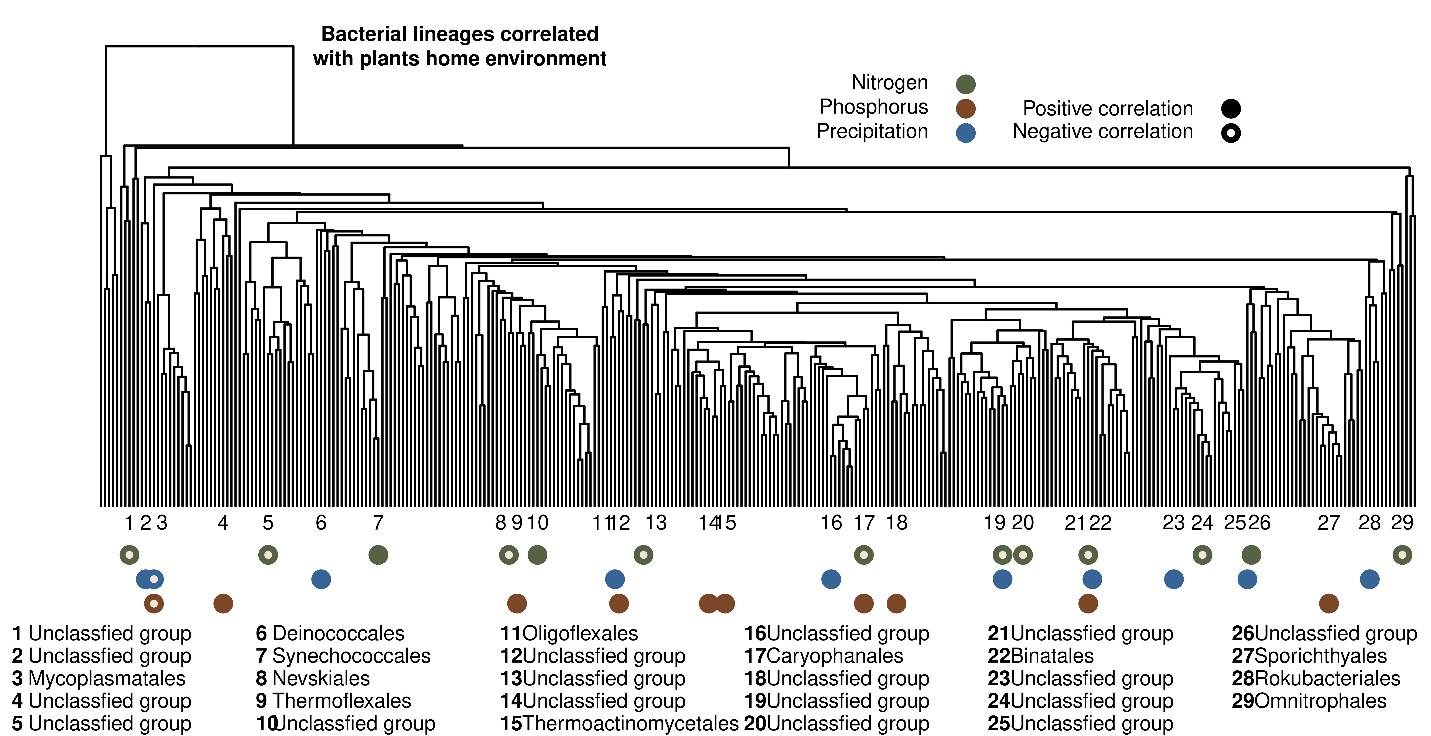


**Fig. S5**

Significant drivers of enrichment in fungal ITS lineages across *Brachypodium distachyon* genotypes, specifically using the home environment from which plants were collected as predictors. We display the correlations between soil cation exchange capacity (CEC) and precipitation with the enrichment of specific microbial lineages as variables were identified as significant in the dbRDA model. To reduce complexity, ASVs were grouped by the taxonomic order to which they were assigned, and evaluated the significance using ANCOMBC. Lineages with significant correlations are labeled on the phylogeny. The lineages are colored based on which variables were significant drivers, with closed vs open circles denoting whether these were positive vs. negative association.


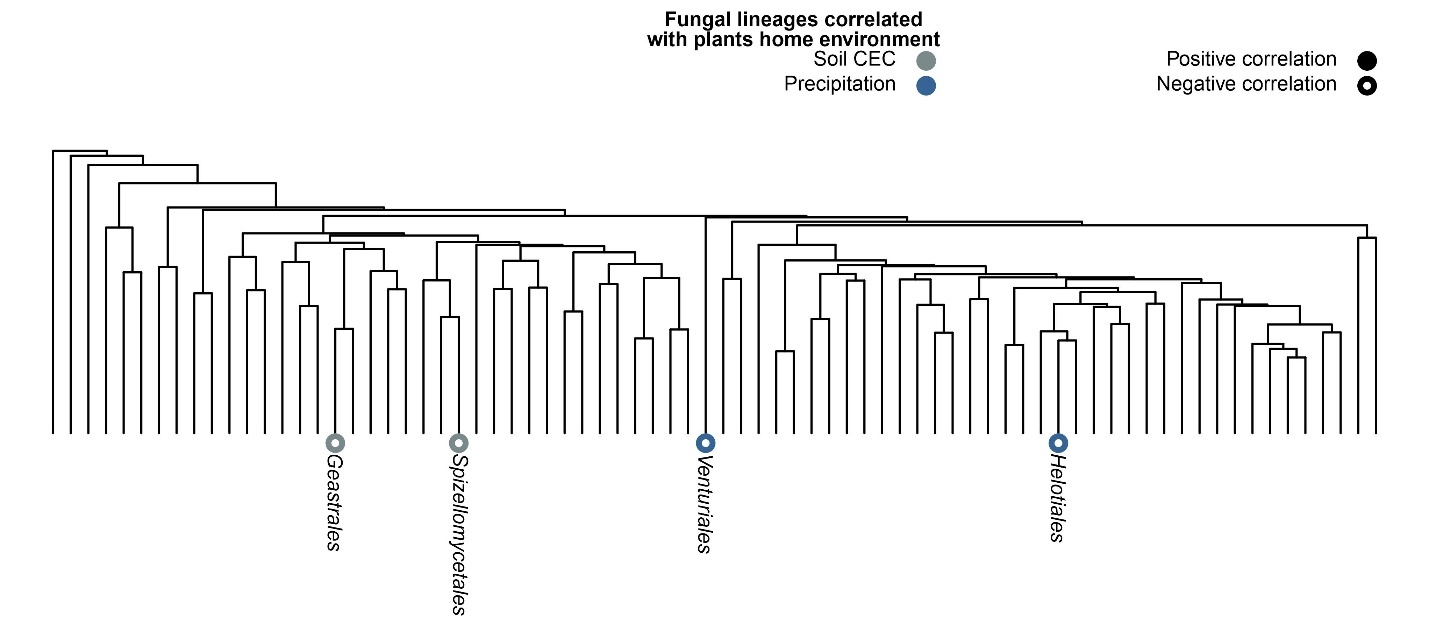


**Fig. S6**

Significant drivers of enrichment in phoD harboring bacterial lineages across *Brachypodium distachyon* genotypes, specifically using the home environment from which plants were collected as predictors. We display the correlations between soil nitrogen and soil phosphorus with the enrichment of specific microbial lineages as variables were identified as significant in the dbRDA model. To reduce complexity, ASVs were grouped by the taxonomic genus to which they were assigned, and evaluated the significance using ANCOMBC. Lineages with significant correlations are labeled on the phylogeny. The lineages are colored based on which variables were significant drivers, with closed vs open circles denoting whether these were positive vs. negative association.


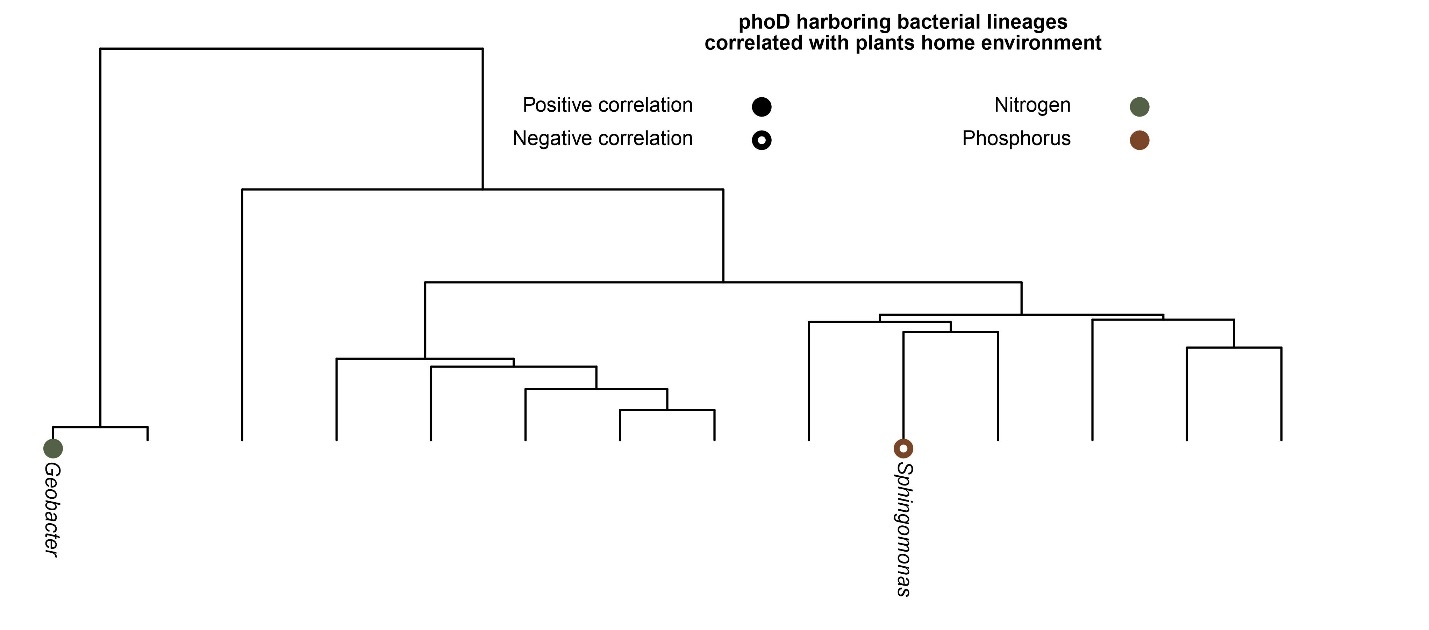


**Fig. S7**

Significant drivers of enrichment in nitrogen-fixing bacterial lineages across *Brachypodium distachyon* genotypes, specifically using the home environment from which plants were collected as predictors. We display the correlations between soil nitrogen with the enrichment of specific microbial lineages as variables were identified as significant in the dbRDA model. To reduce complexity, ASVs were grouped by the taxonomic genus to which they were assigned, and evaluated the significance using ANCOMBC. Lineages with significant correlations are labeled on the phylogeny. The lineages are colored based on which variables were significant drivers, with closed vs open circles denoting whether these were positive vs. negative association.


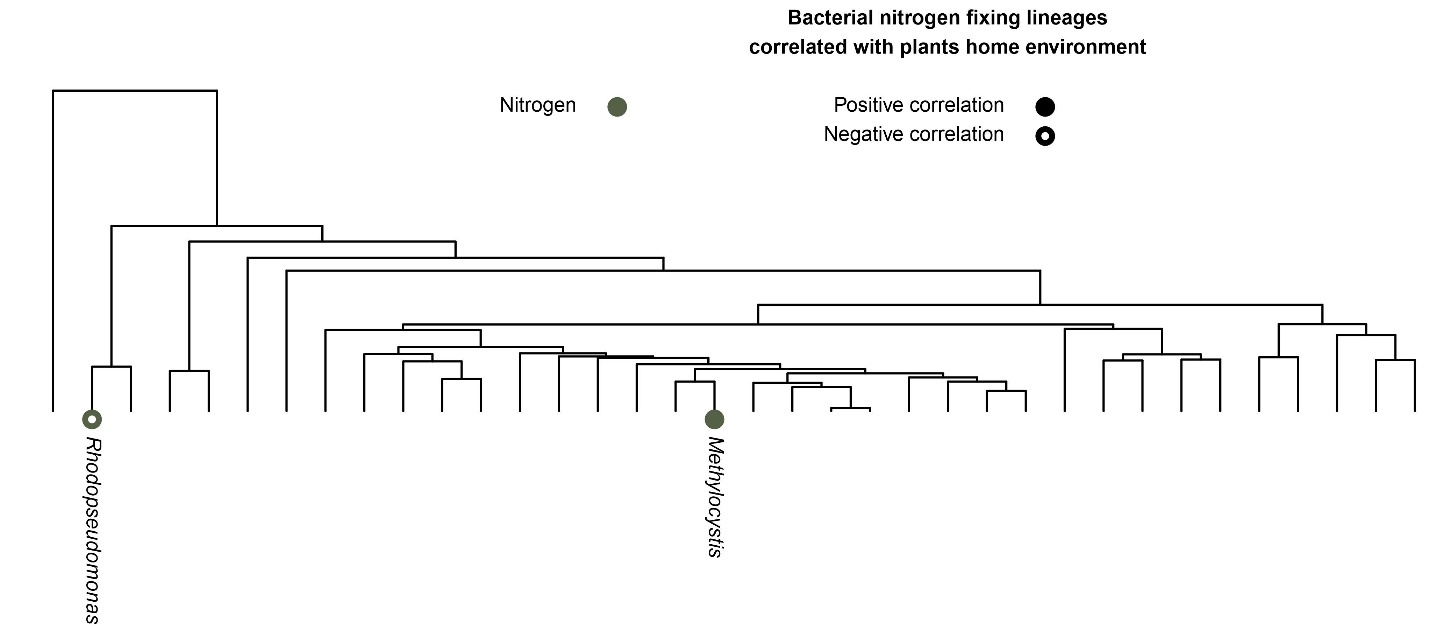


**Fig. S8**

Drivers of bacterial 16S diversity across *Brachypodium distachyon* genotypes, specifically using the home environment from which plants were collected as predictors. We display the correlations between soil nitrogen, soil phosphorus and precipitation, with microbiome diversity metrics, as these variables were identified as significant in the dbRDA model. We feature the correlation between these variables and total richness, evenness (calculated using Pielou’s measure), and the variability among replicates (β dispersion). Each panel has an associated linear mixed effects model associated with in, including the environmental variable as a fixed effect and greenhouse block and plant genotype as random effects. The *p* value for each of these models is displayed in the top right corner of each panel.


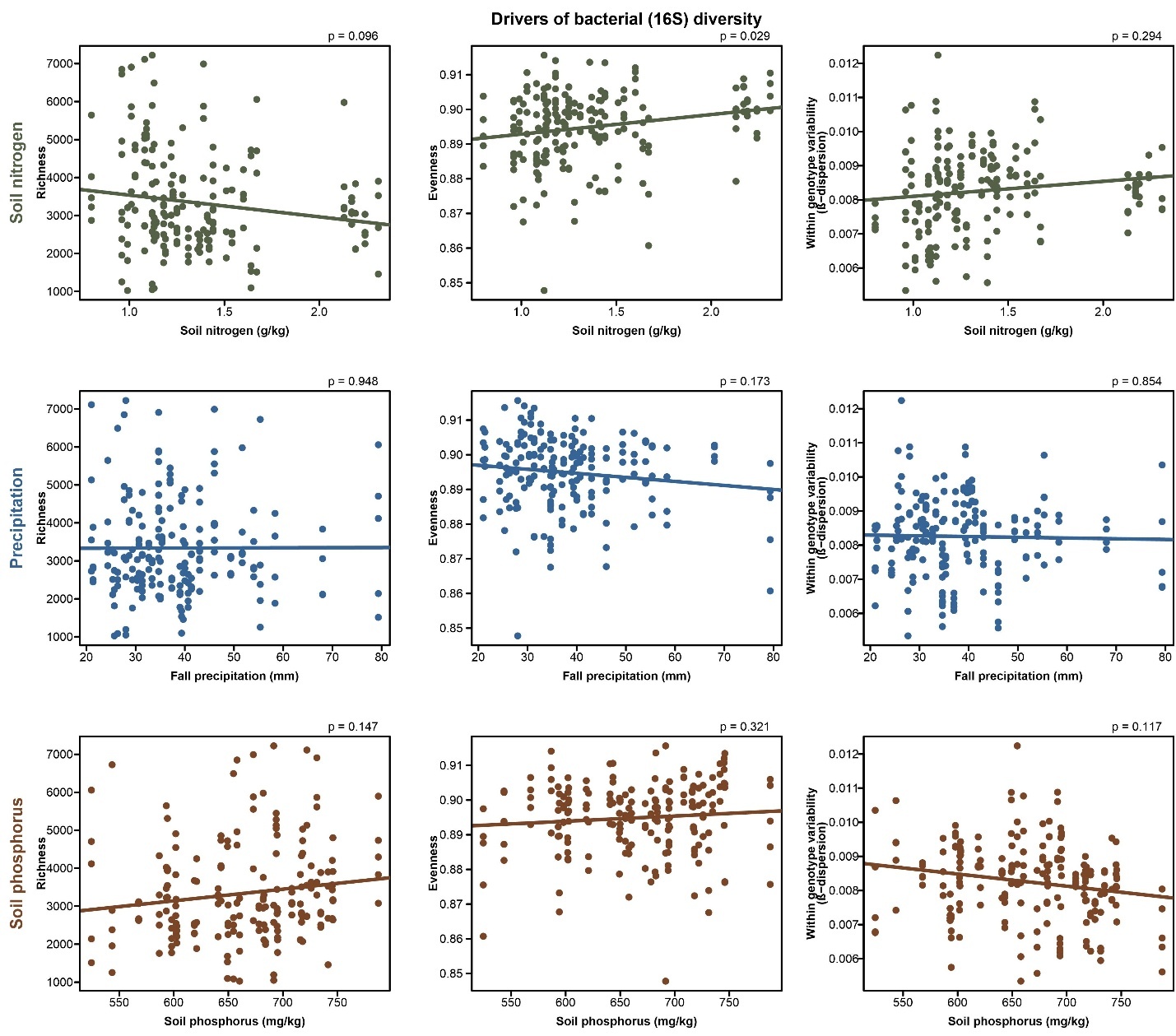


**Fig. S9**

Drivers of fungal ITS diversity across *Brachypodium distachyon* genotypes, specifically using the home environment from which plants were collected as predictors. We display the correlations between precipitation, and soil CEC, with microbiome diversity metrics, as these variables were identified as significant in the dbRDA model. We feature the correlation between these variables and total richness, evenness (calculated using Pielou’s measure), and the variability among replicates (β dispersion). Each panel has an associated linear mixed effects model associated with in, including the environmental variable as a fixed effect and greenhouse block and plant genotype as random effects. The *p* value for each of these models is displayed in the top right corner of each panel.


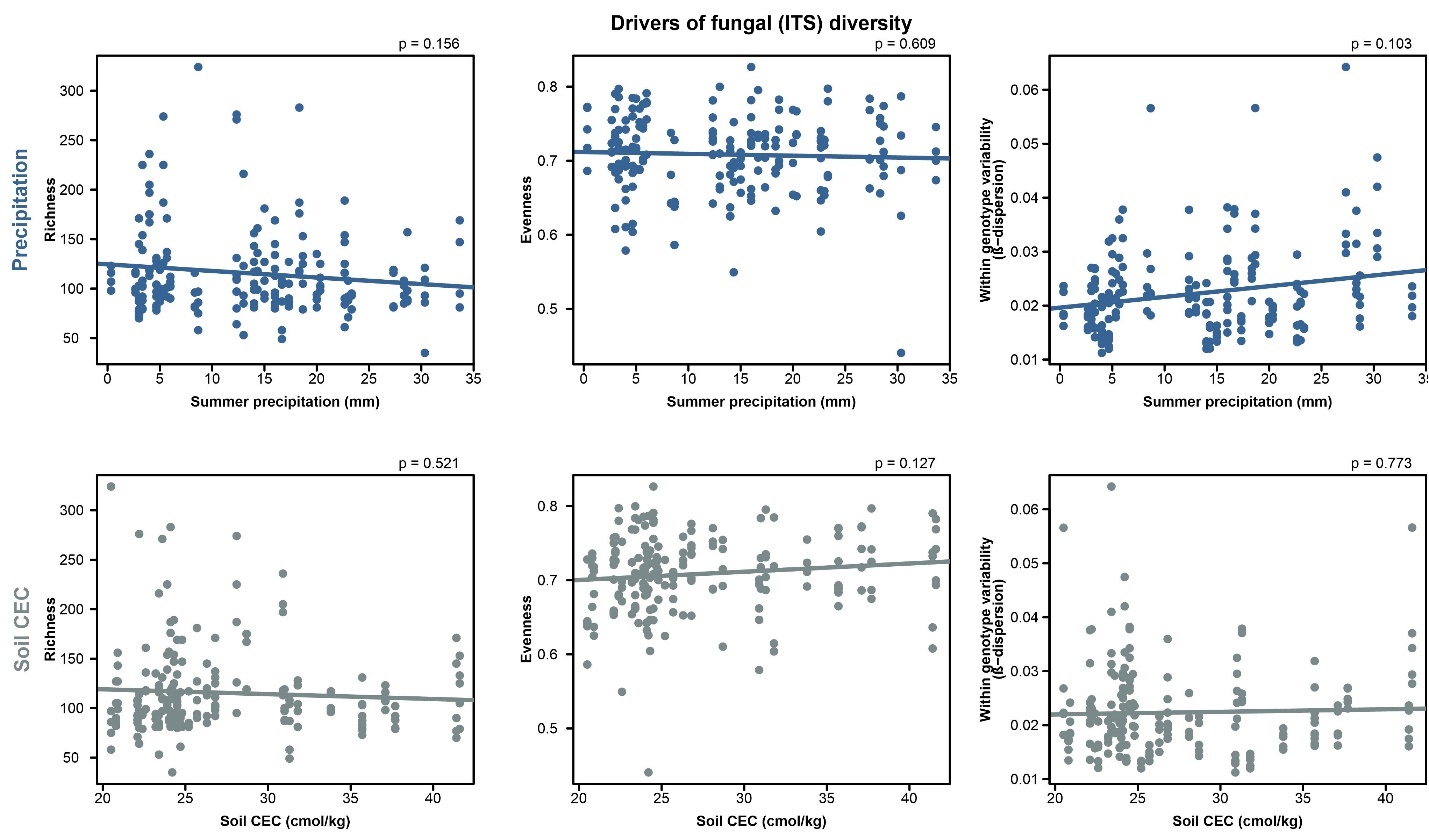


**Fig. S10**

Drivers of AM funal diversity across *Brachypodium distachyon* genotypes, specifically using the home environment from which plants were collected as predictors. We display the correlations between precipitation and elevation, with microbiome diversity metrics, as these variables were identified as significant in the dbRDA model. We feature the correlation between these variables and total richness, evenness (calculated using Pielou’s measure), and the variability among replicates (β dispersion). Each panel has an associated linear mixed effects model associated with in, including the environmental variable as a fixed effect and greenhouse block and plant genotype as random effects. The *p* value for each of these models is displayed in the top right corner of each panel.


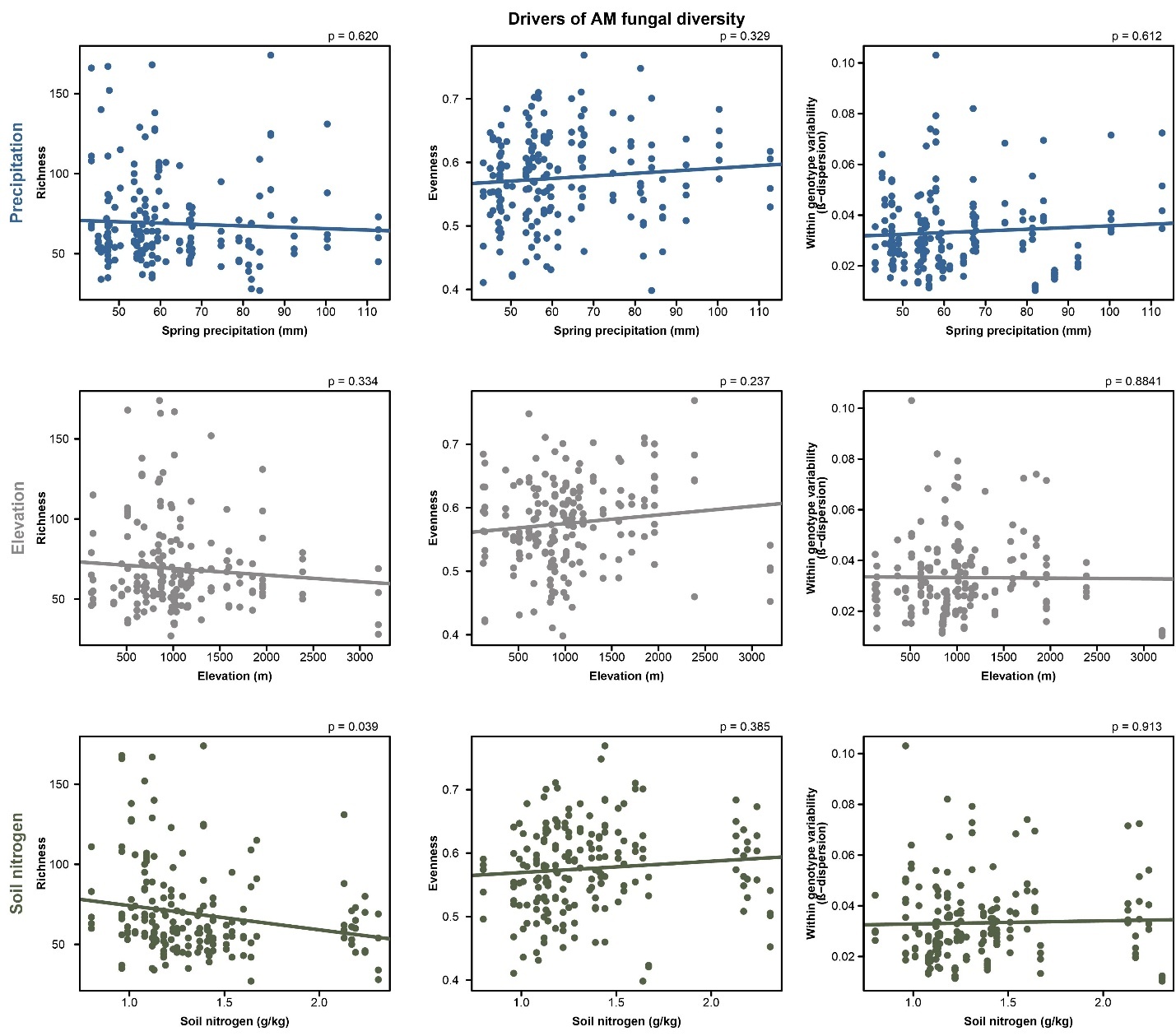


**Fig. S11**

Drivers of phoD harboring bacterial diversity across *Brachypodium distachyon* genotypes, specifically using the home environment from which plants were collected as predictors. We display the correlations between soil nitrogen and soil phosphorus, with microbiome diversity metrics, as these variables were identified as significant in the dbRDA model. We feature the correlation between these variables and total richness, evenness (calculated using Pielou’s measure), and the variability among replicates (β dispersion). Each panel has an associated linear mixed effects model associated with in, including the environmental variable as a fixed effect and greenhouse block and plant genotype as random effects. The *p* value for each of these models is displayed in the top right corner of each panel.


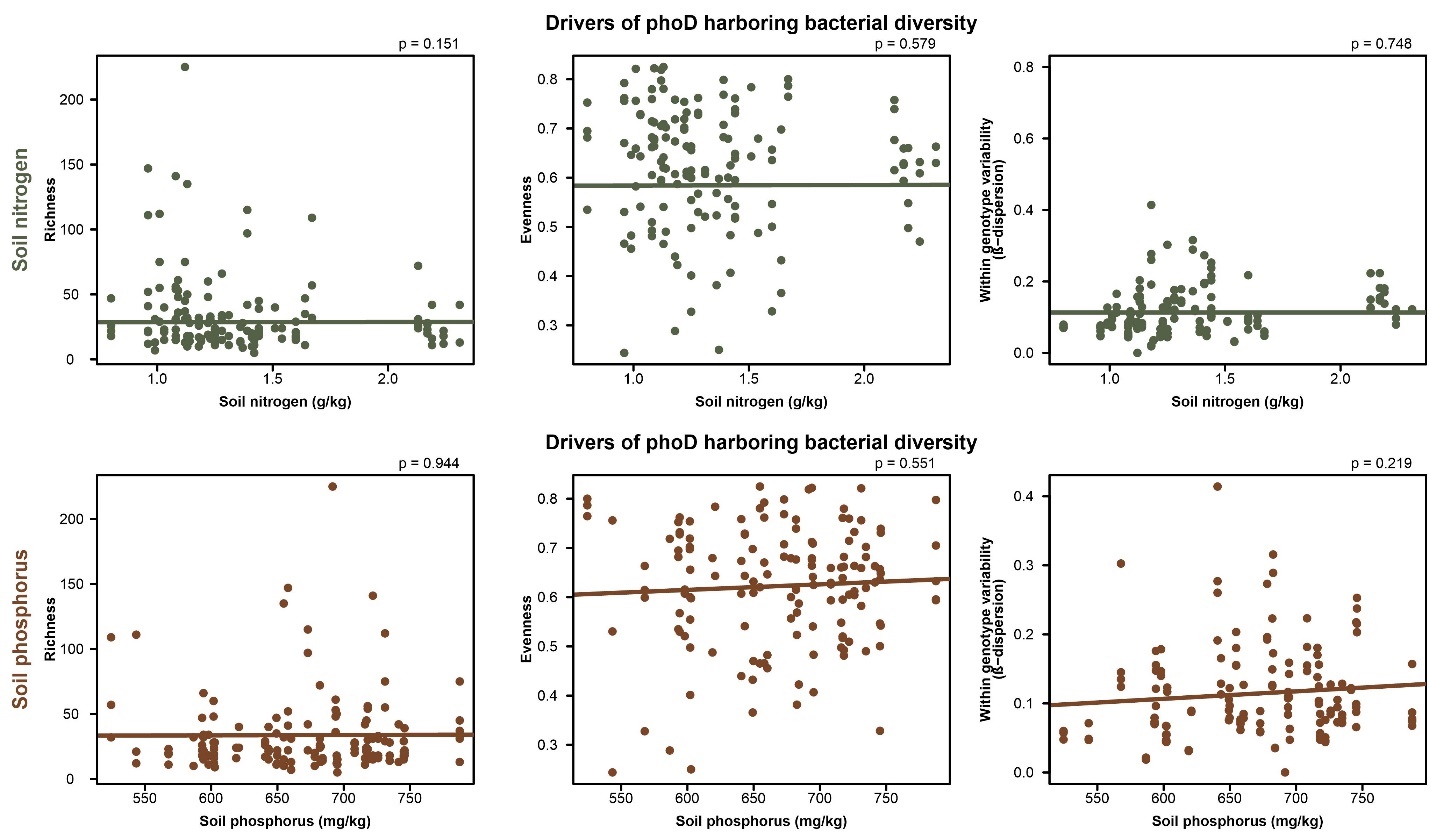


**Fig. S12**

Drivers of ammonia oxidizing microbial diversity across *Brachypodium distachyon* genotypes, specifically using the home environment from which plants were collected as predictors. We display the correlations between soil nitrogen, with microbiome diversity metrics, as these variables were identified as significant in the dbRDA model. We feature the correlation between these variables and total richness, evenness (calculated using Pielou’s measure), and the variability among replicates (β dispersion). Each panel has an associated linear mixed effects model associated with in, including the environmental variable as a fixed effect and greenhouse block and plant genotype as random effects. The *p* value for each of these models is displayed in the top right corner of each panel.


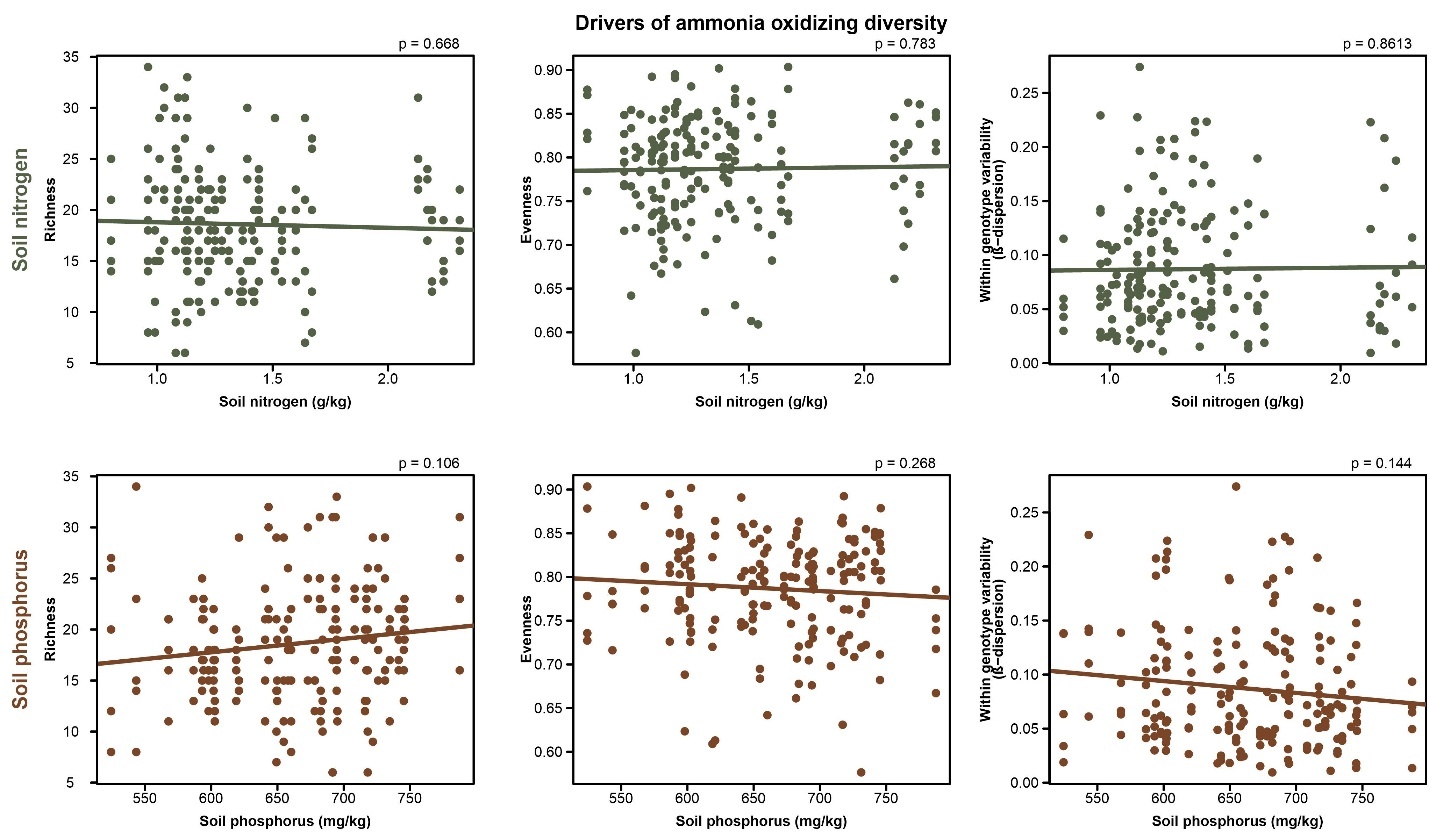


**Fig. S13**

Correlation of nitrogen-fixing bacterial relative abundance with historic soil nitrogen of *Brachypodium distachyon*. We display the correlation with this specific environmental variable, as it was identified as significant in the dbRDA model with this group. Relative abundance was derived from known nitrogen fixers in the 16S dataset. We display an associated linear mixed effects model associated, including the environmental variable as a fixed effect and greenhouse block and plant genotype as random effects. The *p* value for this model is displayed in the top right corner.


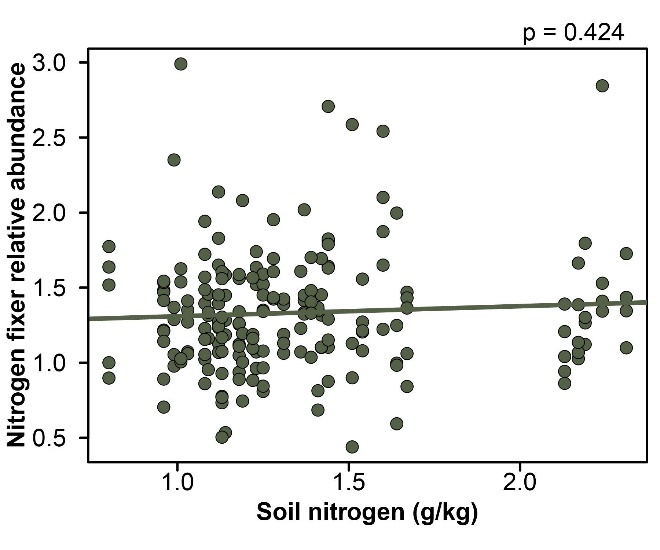


**Fig. S14**

Correlation of AM fungal relative abundance with historic precipitation and elevation of *Brachypodium distachyon*. We display the correlation with these specific environmental variables, as they were identified as significant in the dbRDA model with this group. Relative abundance was derived from glomeromycotan ASVs in the ITS dataset. We display an associated linear mixed effects model associated, including the environmental variables as a fixed effect and greenhouse block and plant genotype as random effects. The *p* value for each of these models is displayed in the top right corner.


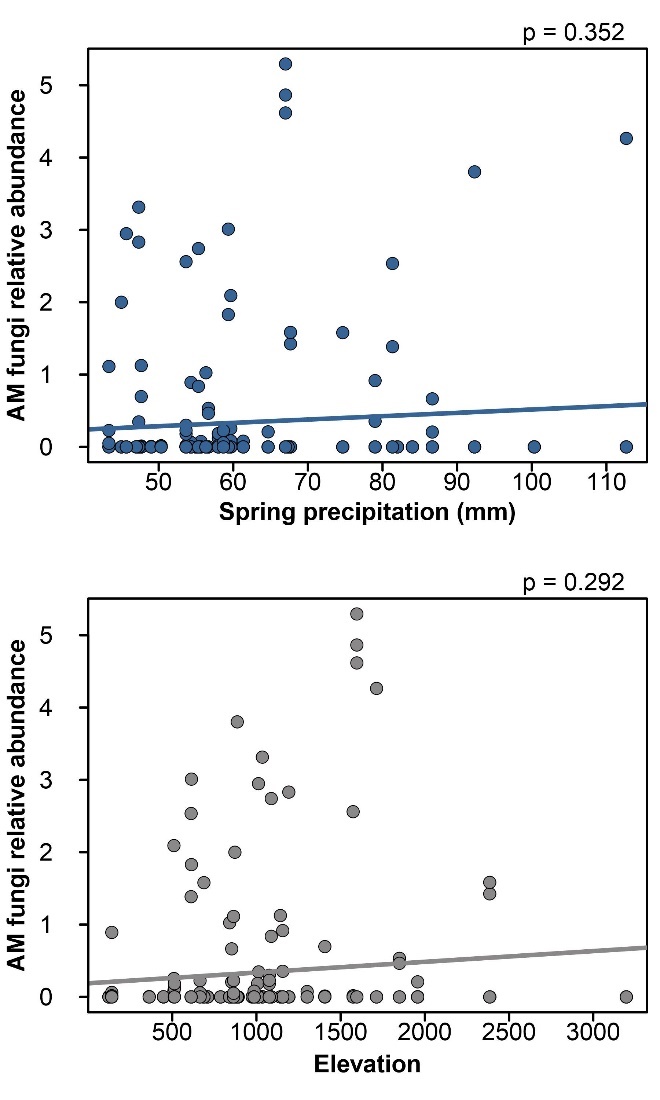


**Fig. S15**

Correlation between nitrogen cycling microbiome composition for each of *Brachypodium distachyon* genotype with plant height and biomass. Data are split to display plant traits when grown in low nitrogen treatments (displayed in orange in the right column), and high nitrogen treatments (displayed in blue in the left column). Points represent the mean estimates of these plant traits for each of the genotypes and are split between when genotypes are grown using soils with live microbes, closed circles, versus with soils using sterile microbes, open circles. Lines in each panel represent the estimated model here, with their corresponding prediction interval. We used nitrogen fixing bacterial richness and ammonia oxidizing bacterial relative abundance as predictor variables.


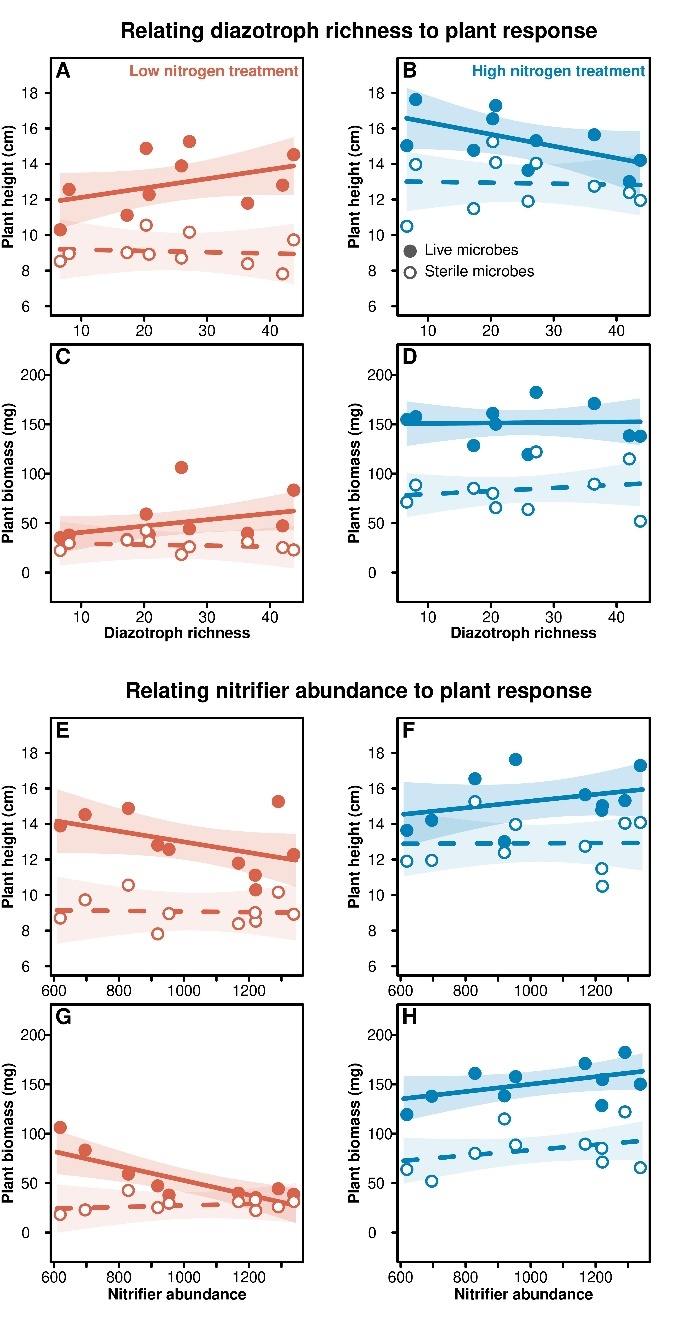


**Table S1**

List of *Brachypodium distachyon* genotypes used in our work. These genotypes were accessed from the USDA GRIN repository, and we included the Accession number here as well as their original collection location.

| **GenotypeID** | **Accession** | **Lat** | **Long** |
| --- | --- | --- | --- |
| G1 | W6 39234 | 37.771 | 38.352 |
| G2 | W6 39270 | 37.128 | 37.391 |
| G3 | W6 39281 | 37.734 | 38.533 |
| G4 | W6 39287 | 38.152 | 41.61 |
| G5 | W6 39301 | 41.085 | 29.315 |
| G6 | W6 39304 | 38.417 | 28.031 |
| G7 | W6 39308 | 38.093 | 28.583 |
| G8 | W6 39311 | 39.087 | 30.252 |
| G9 | W6 39315 | 39.086 | 31.892 |
| G10 | W6 39324 | 38.098 | 34.095 |
| G11 | W6 39325 | 37.768 | 33.52 |
| G12 | W6 39328 | 39.413 | 32.988 |
| G13 | W6 39329 | 38.758 | 34.072 |
| G14 | W6 39331 | 37.109 | 34.071 |
| G15 | W6 39334 | 39.748 | 34.65 |
| G16 | W6 39337 | 37.779 | 35.203 |
| G17 | W6 39338 | 39.748 | 36.816 |
| G18 | W6 39339 | 38.757 | 36.282 |
| G19 | W6 39341 | 37.766 | 37.89 |
| G20 | W6 39342 | 37.435 | 36.819 |
| G21 | W6 39343 | 37.117 | 39.03 |
| G22 | W6 39345 | 37.437 | 40.131 |
| G23 | W6 39346 | 38.095 | 40.659 |
| G24 | W6 39347 | 37.459 | 41.244 |
| G25 | W6 39363 | 40.394 | 32.985 |
| G26 | W6 39371 | 39.754 | 33.538 |
| G27 | W6 39397 | 37.427 | 28.585 |
| G28 | W6 39398 | 39.76 | 29.678 |
| G29 | W6 39404 | 38.43 | 31.309 |
| G30 | W6 39405 | 38.428 | 32.405 |
| G31 | W6 39409 | 37.762 | 39.582 |
| G32 | W6 39413 | 37.798 | 41.774 |
| G33 | W6 39417 | 39.738 | 28.04 |
| G34 | W6 39419 | 41.422 | 27.477 |
| G35 | W6 39423 | 38.097 | 42.322 |
| G36 | W6 39425 | 41.085 | 26.931 |
| G37 | W6 39427 | 36.783 | 32.963 |
| G38 | W6 39428 | 40.406 | 34.636 |
| G39 | W6 39441 | 37.475 | 43.446 |
| G40 | W6 39442 | 36.766 | 44.535 |

**Tables S2**

Primer sets used for the Fluidigm sequencing run. We display the target bacterial group and their associated target gene, as well the primer pairs and a corresponding reference for each.

| **Target group** | **Target gene** | **Primer** | **Forward primer** | **Reverse primer** | **Reference** |
| --- | --- | --- | --- | --- | --- |
| Bacteria | *16S* | 515F/806R | GTGYCAGCMGCCGCGGTAA | GGACTACNVGGGTWTCTAAT | Caporaso et al., 2011;  Parada et al. 2014; |
| Fungi | *ITS2* | 1TS3/ITS4 | GCATCGATGAAGAACGCAGC | TCCTCCGCTTATTGATATGC | White *et al.*, 1990 |
| AM Fungi | *18S* | NS31F/  AMDGR | TTGGAGGGCAAGTCTGGTGCC | CCCAACTATCCCTATTAATCAT | Van Geel *et al.*, 2014 |
| phoD bacteria | *phoD* | PhoDF733/  phoDR1083 | TGGGAYGATCAYGARGT | CTGSGCSAKSACRTTCCA | Ragot *et al.*, 2015 |
| N-fixing bacteria | *nifH* | IGK3/DVV | GCNWTHTAYGGNAARGGNGGNATHGGNAA | ATNGCRAANCCNCCRCANACNACRTC | Ando et al. 2005; Lumini et al. 2010 |

**Tables S3**

Summary statistics for DNA sequencing, displayed for each target microbial group.

| **Target group** | **Target gene** | **Primer** | **Total read** | **Rarefaction depth** | **Samples used** | **Total ASVs** | **Mean ASVs per sample** |
| --- | --- | --- | --- | --- | --- | --- | --- |
| Bacteria | *16S rRNA* | 515F/  806R | 56566794 | 101802 | 193 | 80799 | 3314 |
| Fungi | *ITS2* | ITS2/  ITS3 | 20670005 | 48002 | 194 | 7362 | 115 |
| AM fungi | *18S rRNA* | NS31F/  AMDGR | 9809090 | 22766 | 192 | 3138 | 62 |
| phoD bacteria | *phoD* | phoDF733/  phoDR1083 | 2184596 | 5096 | 141 | 2733 | 33 |
| N-fixing bacteria | *nifH* | IGK3/  DVV | 1236175 | 1253 | 140 | 1440 | 21 |

**Table S4**

Results from dbRDA model correlating community composition, with weighted-Unifrac distances, of each microbial group with the plant genotypes’ home environments. We included the variation explained by each variable chosen in model selection, with each a statistically significant predictor. In models including multiple terms, some of the variation could not be ascribed to one variable alone and instead represented shared variation

| Group | Soil N | Soil CEC | Soil P | Elev. | Soil pH | Temp | Spring precip. | Summer precip. | Fall precip. | Total |
| --- | --- | --- | --- | --- | --- | --- | --- | --- | --- | --- |
| Bacteria | 0.57% | 0% | 0.63% | 0% | 0% | 0% | 0% | 0% | 0.58% | 1.79% |
| Fungi | 0% | 0.53% | 0% | 0% | 0% | 0% | 0.44% | 0% | 0% | 0.96% |
| AM fungi | 0.46% | 0% | 0% | 0% | 0% | 0 % | 0.44% | 0% | 0% | 0.90% |
| *phoD* bacteria | 0% | 0% | 0% | 0% | 0% | 0% | 0% | 0% | 0% | 0% |
| N-fixing bacteria | 2.06% | 0% | 0% | 0% | 0% | 0% | 0% | 0% | 0% | 2.06% |
| Ammonia oxidizers | 0.57% | 0% | 2.09% | 0% | 0% | 0% | 0% | 0% | 0% | 2.69% |

**Table S5**

Results from dbRDA model correlating community composition, with Bray-Curtis distances, of each microbial group with the plant genotypes’ home environments. We included the variation explained by each variable chosen in model selection, with each a statistically significant predictor. In models including multiple terms, some of the variation could not be ascribed to one variable alone and instead represented shared variation

| Group | Soil N | Soil CEC | Soil P | Elev. | Soil pH | Temp | Spring precip. | Summer precip. | Fall precip. | Total |
| --- | --- | --- | --- | --- | --- | --- | --- | --- | --- | --- |
| Bacteria | 0.58% | 0% | 0% | 0% | 0% | 0% | 0% | 0% | 0% | 0.58% |
| Fungi | 0% | 0.55% | 0% | 0% | 0% | 0% | 0% | 0% | 0% | 0.55% |
| AM fungi | 0% | 0% | 0% | 0.66% | 0% | 0% | 65% | 0% | 0% | 1.31% |
| *phoD* bacteria | 0.75% | 0% | 0.87% | 0% | 0% | 0% | 0% | 0% | 0% | 1.62% |
| N-fixing bacteria | 1.02% | 0% | 0% | 0% | 0% | 0% | 0% | 0% | 0% | 1.02% |
| Ammonia oxidizers | 0.84% | 0% | 0% | 0% | 0% | 0% | 0% | 0% | 0% | 0.84% |

**Table S6**

ANOVA tables from mixed effect models, assessing various plant traits, including height, biomass, and tillering status. As fixed effects, models included the abundance of ammonia oxidizing microbes associated with the genotype, the microbe status in greenhouse (live vs sterile soils), the nitrogen status in the greenhouse (high vs low nitrogen treatment), and the interactions between these terms. Biomass and Height were evaluated using normal distributions, and values displayed are *F* statistics for each term. Tillering was evaluated using binomial distributions, and values displayed are χ^2^. Terms with a *p* value less than 0.1 are indicated with the following symbology: + *p* ≤ 0.1; * *p* ≤ 0.05; ** *p* ≤ 0.01; *** *p* ≤ 0.001.

| **Variable** | df | Biomass | Height | Tillering |
| --- | --- | --- | --- | --- |
| Ammonia_Oxidizer_Abundance | 1 | 0.02 | 0.05 | 0.11 |
| Microbe | 1 | 36.40*** | 39.64*** | 6.13* |
| Greenhouse Nitrogen | 1 | 216.62*** | 109.27*** | 22.83*** |
| AmmoOxidAb:Microbe | 1 | 2.22 | 0.12 | 2.24 |
| AmmoOxidAb:GreenhouseN | 1 | 8.81** | 4.86* | 0.15 |
| GreenhouseN:Microbe | 1 | 16.04*** | 5.60* | 0.97 |
| AmmoOxidAb:GreenhouseN:Microbe | 1 | 3.87+ | 3.49+ | 0.09 |
| Residuals | 283 |  |  |  |

**Table S7**

ANOVA tables from mixed effect models, assessing various plant traits, including height, biomass, and tillering status. As fixed effects, models included the richness of nitrogen fixing bacteria associated with the genotype, the microbe status in greenhouse (live vs sterile soils), the nitrogen status in the greenhouse (high vs low nitrogen treatment), and the interactions between these terms. Biomass and Height were evaluated using normal distributions, and values displayed are *F* statistics for each term. Tillering was evaluated using binomial distributions, and values displayed are χ^2^. Terms with a *p* value less than 0.1 are indicated with the following symbology: + *p* ≤ 0.1; * *p* ≤ 0.05; ** *p* ≤ 0.01; *** *p* ≤ 0.001.

| **Variable** | df | Biomass | Height | Tillering |
| --- | --- | --- | --- | --- |
| Nitrogen_Fixer_Richness | 1 | 0.87 | 0.01 | 0.35 |
| Microbe | 1 | 36.83*** | 39.27*** | 6.22* |
| Greenhouse Nitrogen | 1 | 206.68*** | 110.31*** | 21.98*** |
| NFixRich:Microbe | 1 | 0.44 | 0.09 | 0.15 |
| NFixRich:GreenhouseN | 1 | 0.04 | 5.57* | 1.01 |
| GreenhouseN:Microbe | 1 | 15.86 *** | 5.68* | 0.92 |
| NFixRich:GreenhouseN:Microbe | 1 | 1.08 | 5.97* | 0.51 |
| Residuals | 283 |  |  |  |

**Methods S1**

**Correlating microbial effect with historic nitrogen environments**

We characterized potential adaptive nitrogen responses in these microbial effects by correlating the microbial effect terms with their genotype’s associated historic nitrogen environment. Adaptation would be supported with differing reaction norms between the two nitrogen environments. While we could simply use the raw calculated microbial effect for this comparison, all the input live and sterile terms estimates have uncertainty associated with them. We therefore first estimated the uncertainty on our microbial effect terms by propagating error from the Live and Sterile uncertainty estimates input into the term the propagate function in the *propagate* R package (Spiess 2013).

Then, for a given nitrogen environment, we used a simulation approach to estimate the correlation between the microbial effect term and the historic nitrogen environment. Using the estimated uncertainty on our microbial effect terms for each plant genotype, we could simulate data to generate estimates to the strength of the correlation. Each simulated replicate is constructed by generating random estimates for each genotype’s microbial effect, centered on the observed effects with the associated error terms. With each simulated dataset, we could extract a coefficient representing the correlation between the simulated microbial effect terms and the historic nitrogen. This generates a distribution of simulated slope coefficients that represent the confidence of our estimates of this correlation. To compare these estimated slopes between high and low nitrogen environments, we compared the distribution of simulated slopes to extract a *p* value. Significant *p* values suggest the association between the microbial effect and historic nitrogen environment is dependent on the contemporary greenhouse nitrogen treatment.

**REFERENCES**

He X, Augusto L, Goll DS, Ringeval B, Wang Y, Helfenstein J, Huang Y, Yu K, Wang Z, Yang Y, *et al.* 2021. Global patterns and drivers of soil total phosphorus concentration. *Earth System Science Data* 13: 5831–5846.

Spiess A-N. 2013. propagate: Propagation of Uncertainty. 1.0-7.
